# Supplementary material for: Optimization on multifractal loss landscapes explains a diverse range of geometrical and dynamical properties of deep learning
Source: Nat Commun. 2025 Apr 5;16:3252. doi: 10.1038/s41467-025-58532-9 (PMC11971247; doi:10.1038/s41467-025-58532-9)
Supplement: Supplementary file 1 — Supplementary Information [file 41467_2025_58532_MOESM1_ESM.pdf]

Supplementary information for "Optimization on multifractal loss landscapes explains a diverse range of geometrical and dynamical properties of deep learning"

Andrew Ly[1] and Pulin Gong[1*]

[1]School of Physics, University of Sydney, Sydney, NSW, Australia.

*Corresponding author(s). E-mail(s): pulin.gong@sydney.edu.au;

1 Fractional and multifractional Brownian surfaces

We recall results for the fractional and multifractional Brownian surfaces. In the literature, they are commonly known as fractional and multifractional Brownian motions, respectively. However, we generally opt for the former terminology because they they are used in our model for the construction of the loss landscape and, also, to avoid confusion with the "motion" of an optimizer.

The fractional Brownian surface B_H is defined as a centered Gaussian process with covariance function [1–4]:

$$\langle B_H(\boldsymbol{\theta})B_H(\boldsymbol{\theta}')\rangle = \frac{1}{2}(||\boldsymbol{\theta}||^{2H} + ||\boldsymbol{\theta}'||^{2H} - ||\boldsymbol{\theta} - \boldsymbol{\theta}'||^{2H}), \tag{S1}$$

where the Hölder exponent $H \in (0, 1)$ is constant and $\boldsymbol{\theta}, \boldsymbol{\theta}' \in \mathbb{R}^n$. The multifractional Brownian surface is a generalization of the fractional Brownian surface such that its regularity has spatial dependence. The multifractional Brownian surface B_H is a centered Gaussian process with covariance function:

$$\langle B_H(\boldsymbol{\theta})B_H(\boldsymbol{\theta}')\rangle = D_n(H(\boldsymbol{\theta}), H(\boldsymbol{\theta}'))\left(||\boldsymbol{\theta}||^{H(\boldsymbol{\theta})+H(\boldsymbol{\theta}')} + ||\boldsymbol{\theta}'||^{H(\boldsymbol{\theta})+H(\boldsymbol{\theta}')} - ||\boldsymbol{\theta} - \boldsymbol{\theta}'||^{H(\boldsymbol{\theta})+H(\boldsymbol{\theta}')}\right), \tag{S2}$$

where $\boldsymbol{\theta}, \boldsymbol{\theta}' \in \mathbb{R}^n$, $H : \mathbb{R}^n \to (0, 1)$ and $D_n : \mathbb{R} \times \mathbb{R} \to \mathbb{R}$ is a deterministic function. For instance,

$$D_1(x, y) = \frac{\sqrt{\Gamma(2x + 1)\Gamma(2y + 1)\sin(\pi x)\sin(\pi y)}}{2\Gamma(x + y + 1)\sin(\pi(x + y)/2)} \tag{S3}$$

for the 1-D multifractional Brownian surface.

An important property of the multifractional Brownian surface B_H is its local asymptotic self-similarity [5]:

$$\lim_{\varepsilon\to 0^+}\left(\frac{B_H(\boldsymbol{\theta}+\varepsilon\boldsymbol{\theta}')-B_H(\boldsymbol{\theta})}{\varepsilon^{H(\boldsymbol{\theta})}}\right)_{\boldsymbol{\theta}'\in\mathbb{R}^n}\overset{(\mathrm{d})}{=}\left(B_{H(\boldsymbol{\theta})}(\boldsymbol{\theta}')\right)_{\boldsymbol{\theta}'\in\mathbb{R}^n}\ \text{for any}\ \boldsymbol{\theta}\in\mathbb{R}^n, \tag{S4}$$

where the equality is up to a multiplicative deterministic function. The local asymptotic self-similarity roughly means that a dilation or scaling of B_H about any point $\boldsymbol{\theta}$ increasingly resembles the fractional Brownian motion $B_{H(\boldsymbol{\theta})}$ as the scale factor grows.

2 Fractal and multifractal properties

We now elucidate the notion of multifractality and its applicability to our model. We first consider monofractality for instructive purposes. A set X is called monofractal if its scaling properties can be characterized by a Hausdorff fractal dimension d_H that is larger than the topological dimension d (see e.g., [6] and references therein for definitions). For example, the fractional Brownian surface B_H is a self-similar monofractal with fractal dimension $d_H = n+1-H$. As a result, it obeys the following scaling law: $a^H B_H(\boldsymbol{\theta})$ is identically distributed as $B_H(a\boldsymbol{\theta})$ for a constant a. In this sense, B_H is similar to itself after a scale transformation. Note that monofractality is equivalently a description of the roughness or space-filling capacity of an object. To highlight this geometrical connection, we display several realizations of fractional Brownian surfaces with varying values of H in 1-D and 2-D (Fig. S1).

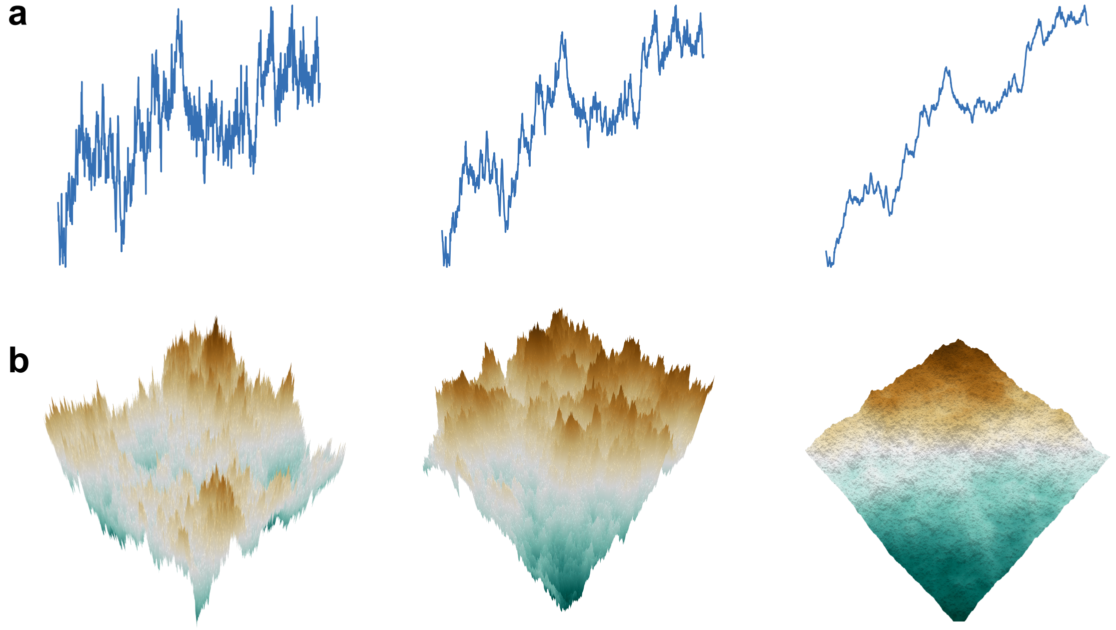

Fig. S1 Increasing the Hölder exponent decreases the roughness of a fractional Brownian surface. a, 1-D fractional Brownian surfaces B_H with global constants $H = 0.3$ (left), $H = 0.5$ (middle) and $H = 0.7$ (right). **b**, Same as **a** but for 2-D. The colour bar is the same as Fig. 1 of the main text.

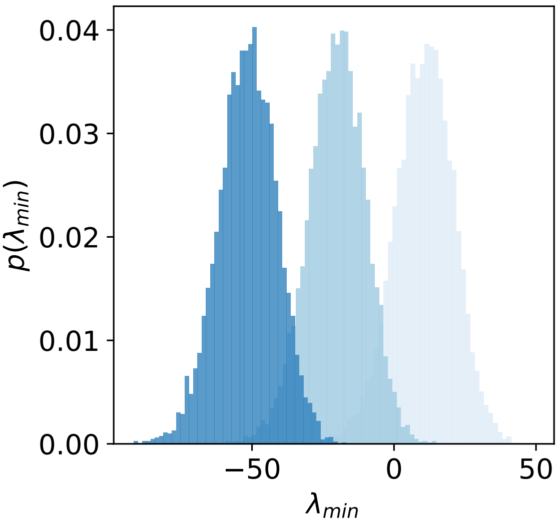

Fig. S2 Empirical distribution of the minimum Hessian eigenvalue. The empirical probability density is calculated using 10^4 random samples of the Hessian matrix for a 10-dimensional loss landscape with Hölder exponent $H = 0.7$ at low, intermediate and high loss values. A darker shade indicates a higher loss.

Multifractality is a generalization of monofractality; it pertains to objects that are described, not by a single scaling exponent, but by a continuous set of scaling exponents. This continuous set is encapsulated by the multifractal singularity spectrum [7], which is defined as:

$$f(\alpha) \equiv d_H(\{\boldsymbol{\theta} : H(\boldsymbol{\theta}) = \alpha\}), \tag{S5}$$

where $H(\boldsymbol{\theta})$ is the pointwise Hölder exponent at $\boldsymbol{\theta} \in \mathbb{R}^n$. For a given α, $f(\alpha)$ is the Hausdorff fractal dimension of the set of points with pointwise Hölder exponent α. A multifractal is also commonly identified by its scaling function $\tau(q)$, where moment $q \in Q$ and Q is an interval on $\mathbb{R}$; the multifractal spectrum $f(\alpha)$ is the Legendre transform of the scaling function $\tau(q)$: $f(\alpha) = \inf_q[q\alpha - \tau(q)]$. The determination of the multifractal singularity spectrum $f(\alpha)$ or scaling function $\tau(q)$ is the focus of multifractal analysis. An object is multifractal if it has a non-trivial multifractal singularity spectrum (i.e., $f(\alpha)$ has wide support) and scaling function (i.e., $\tau(q)$ is nonlinear) [8].

We now relate these concepts to our model. As shown in the main text, the loss landscape L in our model has a wide multifractal singularity spectrum $f(\alpha)$ due to the broad range of pointwise Hölder exponents that coexisting across L. For this reason, we regard the loss landscape L as multifractal. In contrast, the fractional Brownian motion B_H with constant $H \in (0, 1)$ (or a loss landscape constructed from it) is monofractal, since it has a trivial spectrum with $f(H) = 1$ and $f(s) = -\infty$ for all $s \neq H$ by equation (S5). However, rigorously speaking, the loss landscape L is multifractal-like since it is not locally self-similar at all (infinite) scales due to the coarse-graining procedure. Nonetheless, it is locally self-similar over an extended range of scales; although not mathematically precise, this is the sense in which self-similarity is often attributed to objects in nature [9]. For this reason, we use the terms multifractal and multifractal-like interchangeably.

As demonstrated in the main text, the multifractal loss landscape explains many features of true loss landscapes. This includes the global connectivity of solutions

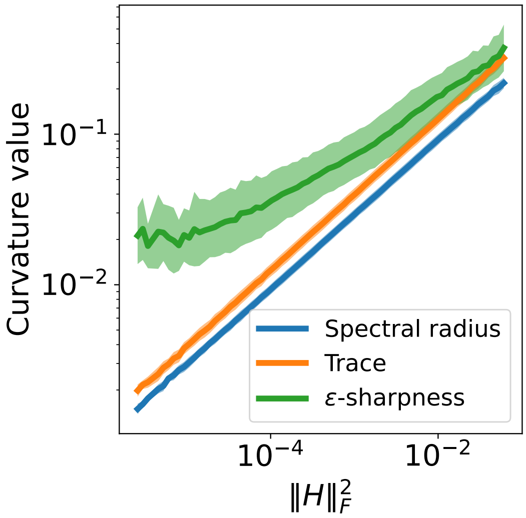

Fig. S3 Comparison of curvature metrics. The spectral radius of the Hessian, trace of the Hessian, and the non-local ϵ-sharpness at all local minima of the multifractal loss landscape. For comparison, these various curvature metrics are plotted against the Frobenius norm. The curves denote the mean values in 100 logarithmically spaced bins. The shaded region contains 2 standard deviations. Note that the ends of the curves are affected by noise due to a low number of data points.

[10, 11] where the smoother basins in the multifractal loss landscape support flatter, highly degenerate solutions connected by low-loss paths. Here, we provide empirical evidence suggesting that the multifractal landscape exhibits another property, which is the absence of spurious local minima. To achieve this, we generate random realizations of the Hessian matrix corresponding to points of varying loss. We then determine the minimum eigenvalue for each realization, noting that a positive minimum eigenvalue implies a local minimum. Figure S2 shows that the minimum eigenvalue distribution shifts in the negative direction as the loss increases and, for a sufficiently high loss, all minimum eigenvalues are negative. Thus, local minima are confined to a band of sufficiently low loss. We find that this result holds for all values of the Hölder exponent H. Moreover, this result is consistent with theoretical characterizations of true loss landscapes in deep learning that imply the absence of spurious local minima [12]. In this context, precise analytical results were possible by connecting the loss landscape to the energy landscape of the multi-spin spherical Ising model, enabling an application of the Kac-Rice formula to calculate the expected number of critical points [13]. However, applying this technique in our model is challenging because, first, the random Hessian matrix has correlated entries and, second, further assumptions are required to constrain the loss. In future work, the latter can be implemented by regulating the large-scale structure of the multifractional Brownian surface (e.g., based on a coercive function) [14].

3 Comparison of curvature metrics

Since various curvature metrics are often correlated with generalization, we compare the Frobenius norm to other common curvature metrics to assess their consistency in the context of the multifractal loss landscape. In particular, we evaluate the spectral radius of the Hessian $\lambda_{\max}$ [15], the trace of the Hessian $\text{Tr}(H)$ and the ϵ-sharpness

[16], calculated as:

$$\frac{\max_{\theta' \in B_\epsilon^\infty}(L(\theta') - L(\theta))}{1 + L(\theta)}, \tag{S6}$$

where B_ϵ^∞ is the ϵ-ball in the l^∞ norm. This modification of the sharpness metric in [16], similar to that in [17], adopts the l^∞ norm to consider points within a square of length 2ϵ centered at θ on the Cartesian grid. We also note that this measure of curvature is non-local, contrasting with other local, Hessian-based metrics. Figure S3 shows that all metrics consistently identify flatter minima within the multifractal landscape. To quantify this consistency, we also measure the Spearman rank correlation coefficients, obtaining the values of 0.995, 0.993 and 0.828 for the spectral radius, trace and ϵ-sharpness (with $\epsilon = 5$), respectively. These high correlation values confirm that smoother regions of the multifractal landscape consistently house flatter minima across all examined curvature metrics.

4 The applicability of our theory to real networks

In this section, we illustrate the general applicability of our theory to real networks. First, we determine the robustness of multifractality in realistic loss landscapes to several changes in training configurations. Throughout this analysis, we demonstrate the utility of the multifractal singularity spectrum $f(\alpha)$ as a theoretical index for capturing the multifractal structure effectively. Although it is mathematically challenging to exactly quantify the real landscape structure, a broad spectrum $f(\alpha)$ serves as a reliable indicator of multifractal geometrical structures in real landscapes. Second, applying the criterion of a broad spectrum $f(\alpha)$, we demonstrate through extensive experiments that when the loss landscape exhibits multifractal characteristics, our theory indeed explains the anomalous diffusive dynamics of GD training. While our theory coherently explains a diverse range of training dynamics and geometrical properties of loss landscapes, it also provides motivation for future research, particularly in the refinement of tools for multifractal analysis.

Multifractal structure of true loss landscapes. Here we investigate the robustness of the real loss landscape analysis to various changes in training configuration. Specifically, we train VGG and ResNet networks on the CIFAR-10 and FashionMNIST datasets using both SGD and Adam optimizers, resulting in a total of 8 different training configurations. Here we present analysis of the training configurations whose corresponding loss landscapes exhibited the most distinct qualities. For all experiments, we use a batch size of 64 and a learning rate of 0.001. In experiments with Adam optimization, we use standard decay rates of 0.9 and 0.999 for the first and second moments, respectively. For VGG networks, we use 16 layers for CIFAR-10 (i.e., VGG-16) and 13 layers for FashionMNIST (i.e., VGG-13) to accomodate different input sizes. For ResNet networks, we use 18 layers regardless of the dataset (i.e., ResNet-18). All training configurations achieve reasonable results (Fig. S4). We calculate multifractal singularity spectra for the two-dimensional sections using the wavelet leader multifractal formalism to parallel the multifractal analysis in the main text. To quantify spatial variations in roughness in greater detail, we additionally estimate pointwise Hölder exponents using a direct increment-based method in the FracLab toolbox [18]. It is worth noting that multifractal formalisms do not estimate pointwise Hölder exponents, instead auxiliary quantities are used to calculate singularity spectra [19]. As a result, there is some discrepancy between the estimated pointwise Hölder

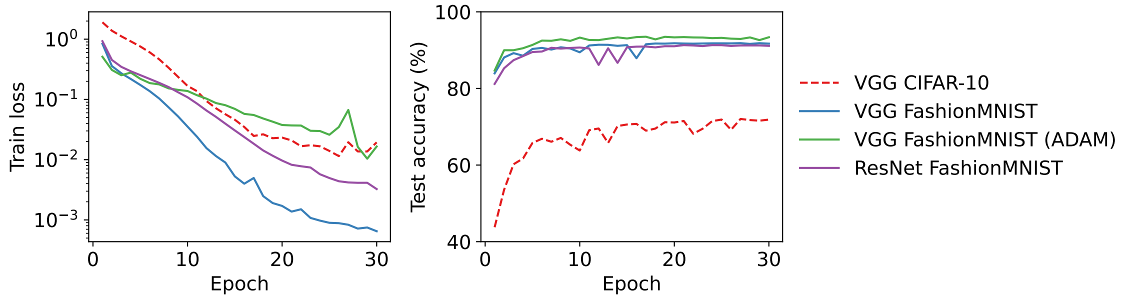

Fig. S4 Learning curves. Cross-entropy loss evaluated on the training dataset and accuracy evaluated on the testing dataset during training for various configurations. Configurations include those corresponding to the loss landscapes in Fig. S5 (solid lines) and the loss landscape in Fig. 3 of the main text (dashed line). A lower accuracy is achieved in the latter as CIFAR-10 classification is a more challenging task than FashionMNIST classification.

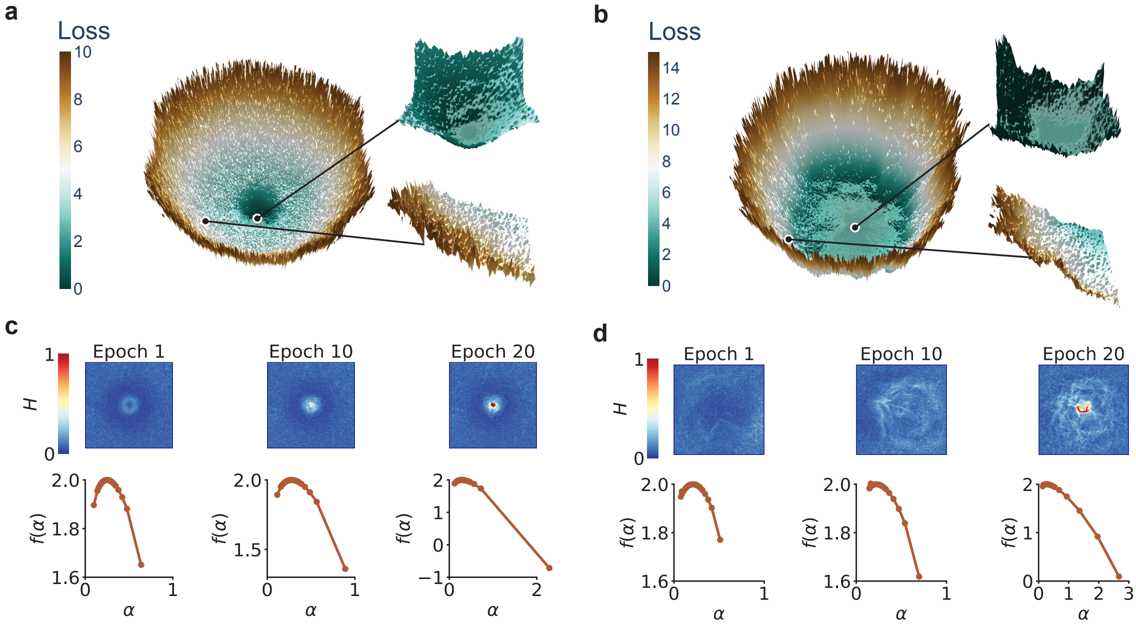

Fig. S5 Loss landscapes of deep neural networks with multifractal structure. a, Visualization of the loss landscape around the optimizer after 20 epochs of training a VGG-13 network on the FashionMNIST dataset using the SGD optimizer. Loss values greater than 10 are clipped. **b**, Similar visualization for a VGG-13 network trained on the FashionMNIST dataset using the Adam optimizer. Loss values greater than 15 are clipped. **c**, The pointwise Hölder exponent (top) and multifractal singularity spectrum (bottom) at various epochs corresponding to the training configuration in **a**. **d**, same as **c** but corresponding to **b**.

exponents and the exponents suggested by the singularity spectra. However, they show qualitatively consistent trends in how roughness changes during training.

We now summarize the main findings of these experiments. Figures S5(a-b) show two-dimensional sections of the loss landscape after 20 epochs of training for a VGG-13 network on FashionMNIST using SGD and a VGG-13 network on FashionMNIST using Adam. These examples correspond to variations in the training data (compared to the main text) and the optimizer, respectively. We observe that multifractal structure is robust to the change from CIFAR-10 to FashionMNIST (Fig. S5(a)) and the change from SGD to Adam (Fig. S5(b)), although the basin is larger in this case. Supporting the findings in the main text, the multifractal spectra $f(\alpha)$ in Figs. S5(c-d) not only capture the presence of multifractal structure (i.e., $f(\alpha)$ is broad), but also

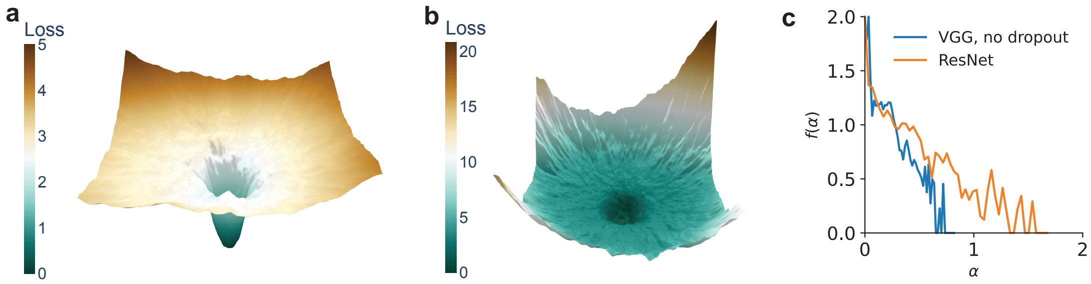

Fig. S6 Loss landscapes of deep neural networks with non-multifractal structure. **a**, Same as Fig. S5(a), except with a ResNet-18 network. **b**, Same as Fig. S5(a), except with the ablation of dropout layers in the VGG-13 network. **c**, Multifractal singularity spectra for these loss landscapes, which are not meaningful due to poor fitting on their non-fractal structures.

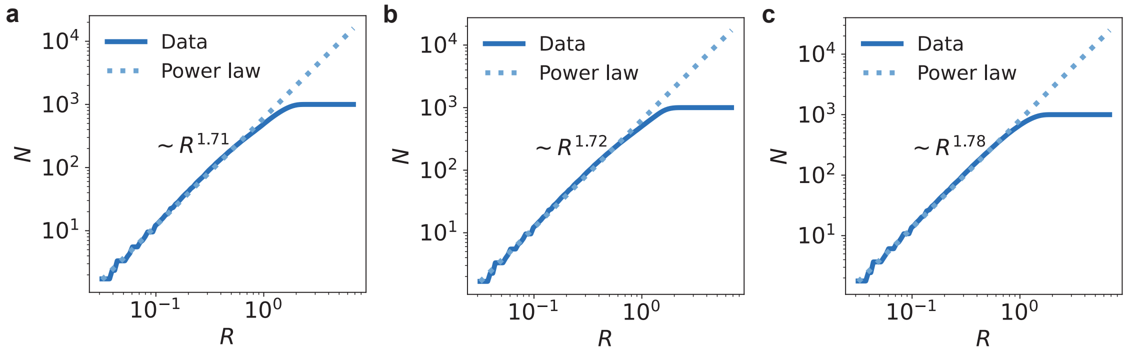

Fig. S7 Clustering in the loss landscape of deep neural networks. Following the clustering analysis of the main text, the 1000 lowest-loss fluctuations within the two-dimensional section of the multifractal loss landscapes are organized as fractal clusters. Their fractal dimension of these clusters is given by the power-law exponents. **a**, VGG-13 network on CIFAR-10 using SGD. **b**, VGG-13 network on FashionMNIST using SGD. **c**, VGG-13 network on FashionMNIST using Adam.

the navigation towards smoother basins (i.e., $f(\alpha)$ broadens to larger α). This is reinforced by the estimations of the pointwise Hölder exponent H, showing increasing H over time in the vicinity of the optimizer.

In contrast, for a ResNet-18 network trained on FashionMNIST using SGD (i.e., the same training configuration as Fig. S5(a), except with a different architecture), we observe that the loss surface is significantly smoother, suggesting the absence of multifractal structure (Fig. S6(a)). In this situation, a spurious multifractal spectrum arises due to poor power-law fitting. Key factors contributing to the smoothness in ResNet landscapes include the presence of residual connections, whose smoothening effects have been empirically shown [20, 21], and the absence of dropout layers. Previous studies have shown that dropout affects the loss landscape and thus optimization dynamics, effects that can be approximated by a stochastic modified equation [22]. Through further ablation analysis, where we apply the same training configuration as Fig. S5(a) except with dropout layers removed, we verify that dropout is indeed a factor contributing to multifractal structure (Fig. S6(b)). Given the widespread use of dropout as a regularization technique in state-of-the-art architectures, multifractal structure similar to Fig. S5(a-b) is expected to be prevalent.

Overall, these findings demonstrate that, as predicted by our theory, the multifractal spectrum $f(\alpha)$ is a robust index for capturing and characterizing the multifractal

nature of real loss landscapes. Specifically, loss landscapes with multifractal characteristics and those without exhibit starkly different multifractal spectra, allowing them to be distinguished. As we demonstrate below, our theory is applicable to the dynamics of GD training in true loss landscapes with multifractal structure as indicated by broad multifractal spectra.

Furthermore, to parallel the fractal clustering analysis in the multifractal model of the main text, we characterize the clustering of points with low loss values compared to surrounding points in the two-dimensional visualizations of the loss landscapes (Fig. S5). We should note that these points are unlikely to correspond to true local minima, especially within higher loss regions, since the high-dimensional loss landscapes of deep networks typically lack spurious local minima [12]. Nonetheless, Figure S7 shows that even the 1000 lowest-loss fluctuations are organized as a fractal cluster for all loss landscapes with multifractal structure. This agrees with the geometric properties of the multifractal loss landscape presented in the main text.

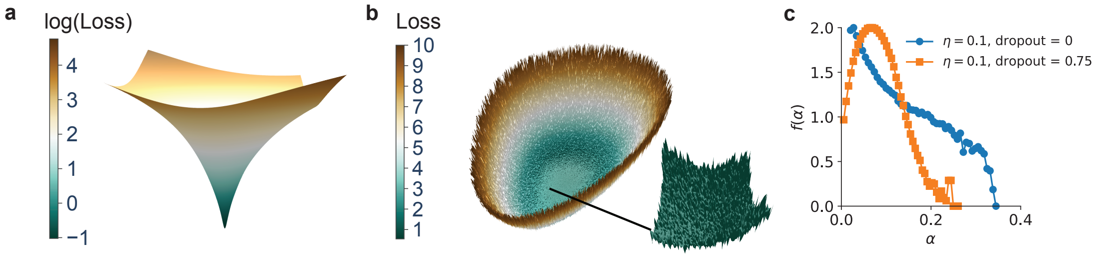

Fig. S8 Loss landscapes in numerical investigation of dynamics. **a**, Visualization of the loss landscape around the GD optimizer ($\eta = 0.1$) after 10^6 epochs of training a 5-16-1 multilayer perceptron on the UCI Airfoil Self-noise dataset [23] without dropout. **b**, Same as **a** but with dropout (0.75). **c**, Comparison of their multifractal spectra. The multifractal spectrum for **b** is the typical shape for a multifractal landscape, resembling an inverted parabola. In contrast, multifractal analysis does not produce a meaningful spectrum for **a** due to poor fitting on a non-fractal smooth landscape.

Training dynamics in true landscapes with multifractal structure. We now demonstrate that when the loss landscape empirically exhibits a multifractal structure, as indicated by a broad multifractal spectrum, then our theory indeed explains the training dynamics. To this end, we analyze the dynamics of GD training in networks with varying levels of dropouts, which, as discussed above, contribute to the multifractal structure. We adopt a similar experimental setup as in [25]. Specifically, we train a fully connected 5-16-1 multilayer network on the UCI Airfoil Self-noise dataset [23] for 10^6 epochs using deterministic GD with MSE loss. We use dropout values of $\{0, 0.25, 0.5, 0.75\}$ and learning rates of $\{10^{-4}, 10^{-3}, 10^{-2}, 10^{-1}, 10^{0}\}$, totaling 20 different training configurations. All configurations with larger learning rates $\eta \geq 10^{-2}$ finish training (i.e., loss plateaus), reaching minimum loss values approximately between 0.1 and 0.5, except for $\eta = 1$ which diverges. In contrast, lower learning rates do not conclude training within 10^6 epochs and generally exhibit higher loss values. We note that the smaller network and dataset used in these experiments, compared to those in Fig. S5 and Fig. S6, enable full-batch GD training for enough epochs to thoroughly characterize the dynamics. Through the same visualization method and spectrum calculation as Fig. S5, we confirm that configurations with dropout exhibit multifractal loss structure, while those without dropout do not (Fig. S8). This further reinforces that dropout is a factor which can contribute to multifractal characteristics.

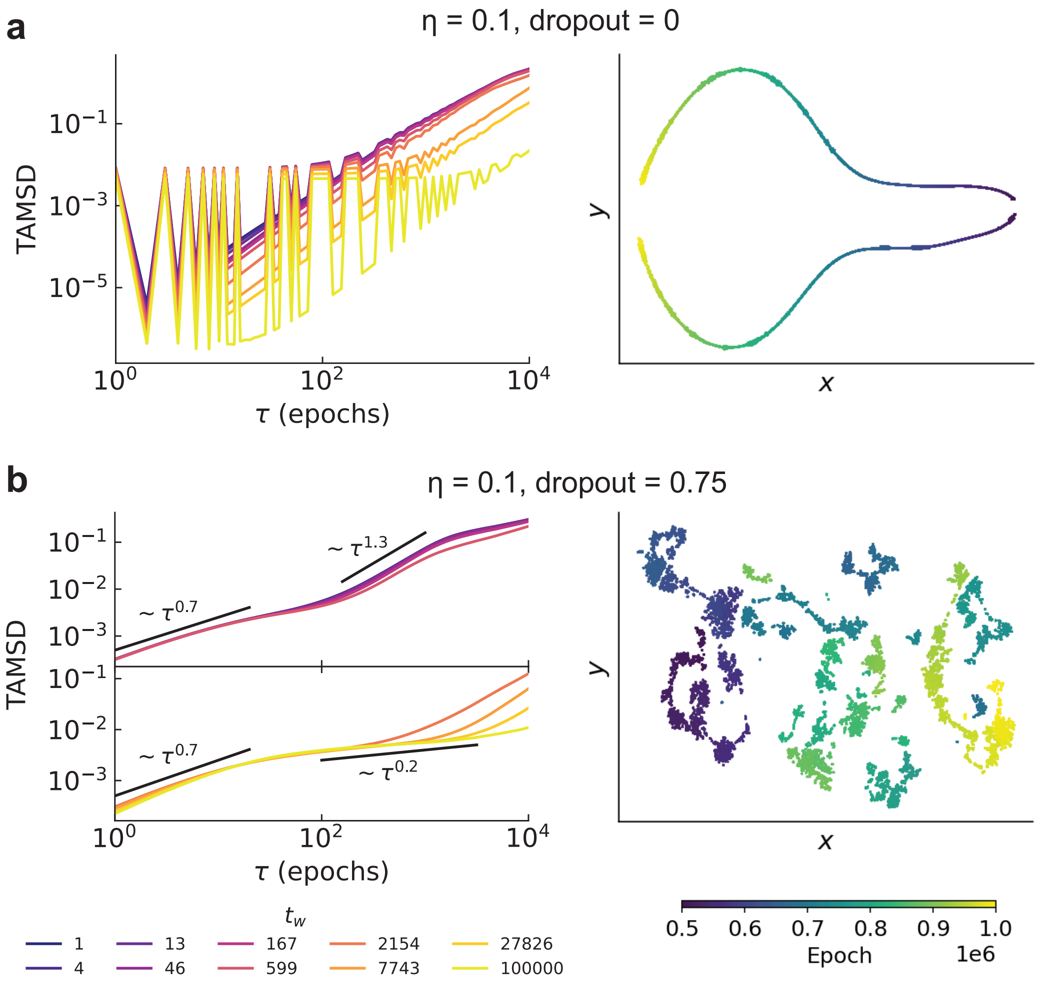

Fig. S9 Dynamics of deterministic GD in real network training. The first column contains TAMSD curves evaluated at logarithmically spaced waiting times with a window size of $T = 10^5$ epochs. Colors represent waiting times, shown in the legend at the bottom. Note that, to reduce the amount of computation, we calculate the TAMSD across logarithmically spaced lag times τ. Eye-guides are provided to indicate approximate diffusion exponents α. The second column shows a visualization of the last 5×10^5 epochs of the GD trajectory using t-SNE dimensionality-reduction [24] with a perplexity value of 500. **a**, Large learning rate ($\eta = 10^{-1}$) and no dropout; the corresponding loss landscape is visualized in Fig. S8(a). The fluctuation of the TAMSD curves at small τ arise from the oscillatory component of the motion. **b**, Large learning rate ($\eta = 10^{-1}$) with dropout (0.75); the corresponding loss landscape is visualized in Fig. S8(b). Similar results occur for other non-zero dropout values.

We now highlight the main findings of these extensive experiments (Fig. S9). For large learning rates without dropout (i.e., no multifractal structure), the dynamics exhibit an oscillatory component (Fig. S9(a)). This is evident from the visualization using t-SNE, a nonlinear dimensionality reduction method [24], showing two separated curves spanning the entire time duration. Thus, despite the high learning rate, there is no anomalous diffusion without multifractal structure. In contrast, when the landscape is multifractal (Fig. S8(b)), non-stationary anomalous diffusion occurs (Fig. S9(b)). Specifically, in early stages of training we find that sub-diffusion ($\alpha \leq 1$) transitions to intermediate super-diffusion ($\alpha > 1$) before ultimately plateauing ($\alpha \to 0$). At late stages of training, sub-diffusion clearly transitions to a plateau. However, while this plateau persists in theoretical TAMSDs derived under the assumption of a harmonic basin (as shown analytically in the main text), in this experiment, it gives way to

further diffusion after an extended duration that increases with the waiting time. Physically, it suggests that confinement in a basin is temporary, lasting longer later into training, and is followed by transition to another basin. This is reinforced by the t-SNE trajectory, revealing spatially separated segments of diffusive motion. We also note that for sufficiently small learning rates, independent of the dropout value (i.e., whether the loss landscape exhibits multifractal structure or not), GD optimization exhibits stable convergence to a local minimum as suggested in the main text. That is, the optimizer descends the loss landscape along curves of monotonically decreasing loss.

In summary, we have demonstrated that our results accurately predict the anomalous diffusive dynamics of GD training when the loss landscape exhibits multifractal structure. Conversely, we have also shown that our theory does not apply when the landscape is smooth. Taken together, the experimental results affirm the applicability of our analysis in realistic training scenarios that meet verifiable criteria based on the multifractal spectrum. As demonstrated in the main text and discussed above, our theory provides a coherent explanation that links a diverse set of geometrical signatures of loss landscapes to various optimization dynamics. Our work thus paves the way for further exploration of the relationships among key factors such as data and architectural influences, the geometry of the loss landscape, and gradient-based training dynamics.

5 Lyapunov exponents

Before introducing Lyapunov exponents, we clarify the notation for this section: We use $M_{ij}^{(t)}$ to represent the ij-entry of the time-dependent matrix $\mathbf{M}^{(t)}$ at time t. Both Lyapunov exponents and Hessian eigenvalues are represented by the symbol λ; to distinguish between them, we subscript Hessian eigenvalues to represent their ordering (e.g., λ_1 is the leading Hessian eigenvalue).

Consider a discrete-time dynamical system $\boldsymbol{\theta}^{(t+1)} = f(\boldsymbol{\theta}^{(t)})$ where f is the evolution rule. We also define the flow map $f^t \equiv f \circ \cdots \circ f$ (t times) such that $\boldsymbol{\theta}^{(t)} = f^t(\boldsymbol{\theta}^{(0)})$. Given a trajectory with initialization $\boldsymbol{\theta}^{(0)}$ and a second trajectory with infinitesimal perturbation $\boldsymbol{\theta}^{(0)} + \delta\boldsymbol{\theta}^{(0)}$, the displacement vector $\delta\boldsymbol{\theta}^{(t)} \equiv f^t(\boldsymbol{\theta}^{(0)} + \delta\boldsymbol{\theta}^{(0)}) - f^t(\boldsymbol{\theta}^{(0)})$ evolves over time according to the linear variational equations (in matrix form):

$$\delta\boldsymbol{\theta}^{(t+1)} = \mathbf{A}(\boldsymbol{\theta}^{(t)})\delta\boldsymbol{\theta}^{(t)}, \tag{S7}$$

where $\mathbf{A}(\boldsymbol{\theta})$ is the stability matrix with entries $A_{ij}(\boldsymbol{\theta}) = \partial f_i(\boldsymbol{\theta})/\partial\theta_j$. Thus, the stability matrix characterizes the local rate of shearing of the infinitesimal neighborhood of $\boldsymbol{\theta}$. Additionally, Taylor expanding the flow to linear order produces:

$$\delta\boldsymbol{\theta}^{(t)} = \mathbf{J}^t(\boldsymbol{\theta}^{(0)})\delta\boldsymbol{\theta}^{(0)}, \tag{S8}$$

where $\mathbf{J}^t(\boldsymbol{\theta}^{(0)})$ is the Jacobian matrix of the finite time flow map with entries $\mathbf{J}_{ij}^t(\boldsymbol{\theta}^{(0)}) = \partial f_i^t(\boldsymbol{\theta}^{(0)})/\partial\theta_j^{(0)} = \partial\theta_i^{(t)}/\partial\theta_j^{(0)}$.

As in the main text, the maximal Lyapunov exponent (MLE) can be computed by:

$$\text{MLE} \equiv \lambda \equiv \lim_{t\to\infty} \frac{1}{t} \ln \frac{||\delta\boldsymbol{\theta}^{(t)}||}{||\delta\boldsymbol{\theta}^{(0)}||} = \lim_{t\to\infty} \frac{1}{2t} \ln(\hat{\mathbf{n}}^T \mathbf{J}^{tT} \mathbf{J}^t \hat{\mathbf{n}}), \tag{S9}$$

where the last equality follows by equation (S8). Here $\hat{n} \equiv \delta\theta^{(0)}/||\delta\theta^{(0)}||$ is a unit vector in the direction of the initial perturbation. The finite-time Lyapunov exponent can be calculated by dropping the limit:

$$\lambda(\boldsymbol{\theta}^{(0)}, \hat{\mathbf{n}}; t) = \frac{1}{2t} \ln(\hat{\mathbf{n}}^T \mathbf{J}^{tT} \mathbf{J}^t \hat{\mathbf{n}}), \tag{S10}$$

which depends on the initialization $\boldsymbol{\theta}^{(0)}$ and the direction of the perturbation $\hat{\mathbf{n}}$. A spectrum of finite-time Lyapunov exponents emerge by considering the spectrum of singular values of $\mathbf{J}^t$. By the polar decomposition theory, we can express the Jacobian $\mathbf{J}^t$ as the product of rotation and stretch matrices: $\mathbf{J}^t = \mathbf{RU}$, where $\mathbf{R}$ is proper orthogonal and $\mathbf{U}$ is symmetric positive definite. The singular values σ_i of $\mathbf{J}^t$ are the eigenvalues of the stretch matrix $\mathbf{U}$: $\mathbf{U}\mathbf{u}_i = \sigma_i \mathbf{u}_i$, where eigenvectors $\mathbf{u}_i$ form an orthonormal basis. A perturbation in the direction $\hat{\mathbf{n}} = \mathbf{u}_i$ thus corresponds to a finite-time Lyapunov exponent of:

$$\lambda(\boldsymbol{\theta}^{(0)}, \mathbf{u}_i; t) = \frac{1}{t} \ln \sigma_i(\boldsymbol{\theta}^{(0)}; t). \tag{S11}$$

To characterize local chaos, we consider the local Lyapunov exponents $\lambda(\boldsymbol{\theta}^{(t)})$, calculated by changing the initialization $\boldsymbol{\theta}^{(0)}$ to $\boldsymbol{\theta}^{(t)}$ and setting the finite time t to 1 iteration in the finite-time Lyapunov exponents (equation (S10)):

$$\lambda(\boldsymbol{\theta}^{(t)}, \hat{\mathbf{n}}) \equiv \lambda(\boldsymbol{\theta}^{(t)}, \hat{\mathbf{n}}; 1) = \frac{1}{2} \ln(\hat{\mathbf{n}}^T \mathbf{J}^1(\boldsymbol{\theta}^{(t)})^T \mathbf{J}^1(\boldsymbol{\theta}^{(t)}) \hat{\mathbf{n}}) = \frac{1}{2} \ln(\hat{\mathbf{n}}^T \mathbf{A}(\boldsymbol{\theta}^{(t)})^T \mathbf{A}(\boldsymbol{\theta}^{(t)}) \hat{\mathbf{n}}), \tag{S12}$$

where $\mathbf{A}(\boldsymbol{\theta}^{(t)})$ is the stability matrix. The local Lyapunov exponents thus characterize the local expansion and contraction of the displacement vector between two trajectories, according to equation (S7). For the gradient descent algorithm, where the evolution rule is $\boldsymbol{\theta}^{(t+1)} = f(\boldsymbol{\theta}^{(t)}) = \boldsymbol{\theta}^{(t)} - \eta \nabla L(\boldsymbol{\theta}^{(t)})$, the stability matrix is $\mathbf{A}(\boldsymbol{\theta}^{(t)}) = \mathbf{I} - \eta \mathbf{H}(\boldsymbol{\theta}^{(t)})$, where $\mathbf{H}$ is the Hessian matrix.

We now analyze the local chaotic behavior of GD. We note that the conditions of local chaos have been derived in a previous study [26]; we review it to make explicit the link to the edge of stability. For each $(\lambda_i, \mathbf{v}_i)$ eigenpair of the Hessian matrix $\mathbf{H}(\boldsymbol{\theta}^{(t)})$, $(1 - \eta\lambda_i, \mathbf{v}_i)$ is an eigenpair of the stability matrix $\mathbf{A}(\boldsymbol{\theta}^{(t)})$. Since the eigenvectors $\mathbf{v}_i$ form an orthonormal basis, we can write a normalized perturbation $\hat{\mathbf{n}}$ as the linear combination $\hat{\mathbf{n}} = \sum_i (\mathbf{v}_i \cdot \hat{\mathbf{n}}) \mathbf{v}_i$. Hence, we can understand local chaotic behavior for an arbitrary perturbation in terms of the local Lyapunov exponent in the eigenvector directions. Given $\hat{\mathbf{n}} = \mathbf{v}_i$, then the local Lyapunov exponent is $\lambda(\boldsymbol{\theta}^{(t)}, \mathbf{v}_i) = \frac{1}{2} \ln(1 - \eta\lambda_i)^2$. Thus, a perturbation along the $\mathbf{v}_i$ direction is:

$$\begin{cases} \text{expanding if } \lambda(\boldsymbol{\theta}^{(t)}, \mathbf{v}_i) > 0, \text{ i.e., } \lambda_i < 0 \text{ or } \lambda_i > 2/\eta \\ \text{marginal if } \lambda(\boldsymbol{\theta}^{(t)}, \mathbf{v}_i) = 0, \text{ i.e., } \lambda_i = 0 \text{ or } \lambda_i = 2/\eta \\ \text{contracting if } \lambda(\boldsymbol{\theta}^{(t)}, \mathbf{v}_i) < 0, \text{ i.e., } 0 < \lambda_i < 2/\eta. \end{cases} \tag{S13}$$

Note that we identify $\lambda(\boldsymbol{\theta}^{(t)}, \mathbf{v}_i) = -\infty$ when $\lambda_i = 1/\eta$, corresponding to the maximally contracting case where $\mathbf{A}(\boldsymbol{\theta}^{(t)})\delta\boldsymbol{\theta}^{(t)} = 0$. Therefore, the GD trajectory is locally chaotic (in some direction corresponding to an eigenvector of the Hessian matrix $\mathbf{H}$)

if $\lambda_i < 0$ or $\lambda_i > 2/\eta$. A sufficient condition for this is that the leading Hessian eigenvalue satisfies $\lambda_1 > 2/\eta$. Thus, the stability threshold (separating stable and unstable dynamics) coincides with the onset of local chaos in the direction of the leading Hessian eigenvector.

Finally, to link local chaos to global chaos (in the sense of infinite time), we show that the finite-time Lyapunov exponent, and thus the MLE, is simply the time-average of the local Lyapunov exponent [27]. By the chain rule, the Jacobian matrix of the flow map evolves as $\mathbf{J}^t(\boldsymbol{\theta}^{(0)}) = \frac{\partial\boldsymbol{\theta}^{(t)}}{\partial\boldsymbol{\theta}^{(0)}} = \frac{\partial\boldsymbol{\theta}^{(t)}}{\partial\boldsymbol{\theta}^{(t-1)}}\frac{\partial\boldsymbol{\theta}^{(t-1)}}{\partial\boldsymbol{\theta}^{(0)}} = \mathbf{A}(\boldsymbol{\theta}^{(t-1)})\mathbf{J}^{t-1}(\boldsymbol{\theta}^{(0)})$. Hence, we can express the finite-time Lyapunov exponent as:

$$\lambda(\boldsymbol{\theta}^{(0)}, \hat{\mathbf{n}}; t) = \frac{1}{2t}\ln(\hat{\mathbf{n}}^T\mathbf{A}(\boldsymbol{\theta}^{(0)})^T \dots \mathbf{A}(\boldsymbol{\theta}^{(t-1)})^T\mathbf{A}(\boldsymbol{\theta}^{(t-1)}) \dots \mathbf{A}(\boldsymbol{\theta}^{(0)})\hat{\mathbf{n}}) \tag{S14}$$

$$= \frac{1}{2t}\left[\ln(\hat{\mathbf{n}}^T\mathbf{A}(\boldsymbol{\theta}^{(0)})^T \dots \mathbf{A}(\boldsymbol{\theta}^{(t-2)})^T\mathbf{A}(\boldsymbol{\theta}^{(t-2)}) \dots \mathbf{A}(\boldsymbol{\theta}^{(0)})\hat{\mathbf{n}}) + 2\lambda(\boldsymbol{\theta}^{(t-1)}, \hat{\mathbf{n}}^{(t-1)})\right] \tag{S15}$$

$$\vdots$$

$$= \frac{1}{t}\sum_{i=0}^{t-1}\lambda(\boldsymbol{\theta}^{(i)}, \hat{\mathbf{n}}^{(i)}), \tag{S16}$$

where $\hat{\mathbf{n}}^{(t)} \equiv \prod_{i=0}^{t-1}\mathbf{A}(\boldsymbol{\theta}^{(i)})\hat{\mathbf{n}}/\|\prod_{i=0}^{t-1}\mathbf{A}(\theta^{(i)})\hat{\mathbf{n}}\|$ is the normalized perturbation after t iterations. Here we have assumed that $\hat{\mathbf{n}}^{(j)} \neq \mathbf{0}$ for $j = 0, 1, \dots, t-1$ (i.e., the perturbation is not maximally contracted at any point in the trajectory). The result shows that the finite-time Lyapunov exponent is the time-average of the local Lyapunov exponent. Taking the infinite-time limit yields the maximal Lyapunov exponent:

$$\lambda = \lim_{t\to\infty}\frac{1}{t}\sum_{i=0}^{t-1}\lambda(\boldsymbol{\theta}^{(i)}, \hat{\mathbf{n}}^{(i)}). \tag{S17}$$

Thus, if, on average, the infinitesimal neighborhood of the GD trajectory is expanding, then it is chaotic. In the main text, we have shown that local chaos indeed extends to global chaos in our model, thus the EoS phenomenon corresponds to an extended edge of chaos.

6 Additional details on the TAMSD

Stochastic motions are commonly analyzed in terms of their mean squared displacement [28]. In theory, it is simpler to analytically derive the ensemble-averaged mean squared displacement, given by:

$$\langle\|\boldsymbol{\theta}(\tau)\|^2\rangle = \int \|\boldsymbol{\theta}\|^2 p(\boldsymbol{\theta}, \tau)\, d\boldsymbol{\theta}, \tag{S18}$$

where $p(\boldsymbol{\theta}, \tau)$ is the probability density function for the particle to be at position $\boldsymbol{\theta}$ at time τ. However, due to experimental constraints, such as the computational cost of conducting many training runs, it is typical to measure the time-averaged mean

squared displacement (TAMSD) instead [28]:

$$\overline{\delta^2}(t_w, \tau) \equiv \frac{1}{T} \sum_{t=t_w}^{t_w+T} \|\boldsymbol{\theta}_{t+\tau} - \boldsymbol{\theta}_t\|^2, \tag{S19}$$

which represents the squared displacement of a particle after a lag time τ with respect to a reference position at the waiting time t_w, time-averaged with a window of size T. In ergodic processes, the ensemble and time averages coincide in the limit of long measurement times.

There are some important practical considerations when analyzing the TAMSD. The TAMSD can reveal additional details about the dynamics that are inaccessible to the ensemble-averaged mean squared displacement (EAMSD) [28]. For instance, the dependence of the TAMSD on the waiting time t_w can illustrate non-stationary aging behavior, which is particularly valuable for characterizing fractional diffusion processes since they are non-Markovian. As demonstrated in the main text, the dynamics of GD in a multifractal loss landscape are indeed non-stationary, transitioning from an initial regime characterized by transient super-diffusion to a purely sub-diffusive regime.

We now provide further physical justification of the anomalous diffusion of GD in a multifractal loss landscape. Recall that the power-law growth of the TAMSD can be expressed as $\overline{\delta^2} \propto \tau^\alpha$, where α is the diffusion exponent. Normal diffusion is associated with $\alpha = 1$. Anomalous diffusion occurs when this is violated, that is, $\alpha \neq 1$. Anomalous diffusion can have various origins [29]: non-stationarity (e.g., Brownian motion with time-changing temperature), extreme events (e.g., Lévy flight with heavy-tailed steps), or temporal correlations (e.g., fractional Brownian motion). The latter is particularly relevant for explaining anomalous diffusion of GD in a multifractal loss landscape, which we model using the overdamped fractional Langevin equation (FLE).

As demonstrated in the main text, the temporal correlations inherent in the FLE lead to sub-diffusion at small lag times τ, regardless of the waiting time t_w, with diffusion exponent $\alpha(H) = \min\{1, 2 - 2H\}$. These temporal correlations arise from spatial correlations associated with the rough structure of the loss landscape, characterized by Hölder exponent H. We theoretically explicate this connection in Supplementary Sec. 8. The overdamped assumption is also important; it implies that the optimizer experiences no inertial effects. Such inertia, which is absent in an optimizer without momentum, typically manifests in the TAMSD as characteristic ballistic motion ($\alpha = 2$) at small τ, preceding sub-diffusion. Without inertia, this initial ballistic phase is absent.

Similarly, the transition to transient super-diffusion at short waiting times t_w arises from correlated steps. Specifically, the bias of the tilted washboard potential (or more general loss structure) drives the optimizer in a particular direction, resulting in the persistence (positive correlation) of steps. For further insight, consider an optimizer in a linear potential (i.e., the periodic component of the tilted washboard is removed). In this idealized example, the steps are perfectly correlated and the optimizer exhibits ballistic motion $x^2(\tau) \sim \tau^2$, that is, $\alpha = 2$. However, the periodic structure on the tilted washboard, or more general non-linear structure in the loss landscape, slows the

otherwise ballistic motion of the optimizer to super-diffusion ($1 < \alpha < 2$). The transition point from sub-diffusion to super-diffusion roughly coincides with the number of iterations needed on average for the optimizer to traverse a periodic fluctuation. We note that the explanation is similar in the underdamped FLE [30], except in this case the particle can exhibit hyper-diffusion ($\alpha > 2$) due to acceleration by the bias. Super-diffusion occurs only for small t_w because the optimizer is eventually trapped in a basin (with infinite escape time for $H > 1/2$), no longer influenced by an overall bias. Similarly, saturation occurs at large τ for all t_w because the optimizer is eventually confined and thus the displacement cannot grow indefinitely.

We note that the features of the TAMSD (e.g., estimates of the diffusion exponent) can vary between individual trials or sample trajectories, called "strange kinetics" [31]. This phenomenon is fundamentally linked to weak ergodicity breaking [32], which describes the situation in which the exploration of single trajectories can become constrained in certain regions of phase space for a long time. Since the resulting exploration times of the phase space is long in these scenarios, single trials of finite duration can yield time-averaged quantities that differ. This effect can be seen for GD in a multifractal loss landscape. In particular, the transient super-diffusion that occurs in the first regime can disappear if the optimizer is initialized within a low-loss basin, as opposed to far from it.

7 TAMSD and flatness

We now derive that the time-averaged displacement of network parameters decreases polynomially with the flatness of a solution as $\overline{\delta^2} \sim F^{-4}$, in the smooth limit where the pointwise Hölder exponent $H \to 1^-$. It is important to note that this illustrates the naturally adaptive behavior of GD learning dynamics in a multifractal landscape, akin to a landscape-dependent annealing process. The derivation relies on two theoretical aspects of our model: First, sub-diffusion is slower in smoother basins according to $\alpha(H) = \min\{1, 2-2H\}$ (equation (14) in the main text). Second, smoother basins are populated by clusters of flatter minima according to $\langle ||\mathbf{H}||_F^2 \rangle \propto r(H)\chi^{2H-2}$, where $r(H) = 16 - 2^{2H+2} + 2^{2H} - 2^{3H-1}$ in 2-D (equation (5) in the main text). The latter illustrates that the expected curvature approaches zero as Hölder exponent H increases to one, since $r(H) \to 0$ as $H \to 1^-$. This is true in arbitrary dimensions; we demonstrate this for 1-D and 2-D in Fig. S10.

The relation we wish to derive is partially motivated by a previous analysis of SGD, which showed an inverse relationship between network parameter variance and flatness [33]:

$$\sigma_i^2 \sim F_i^{-\psi}, \tag{S20}$$

where σ_i^2 is the network parameter variance in the i-th principal component direction, F_i is the corresponding flatness and $\psi \approx 3.8$ empirically [33]. Here, the flatness F_i is defined as the size of the region around a local minimum L_0 in the i-th principal component direction in which the loss value is below $e \times L_0$. Additionally, it was found that the loss function around the local minimum at the end of training a simple neural network on the MNIST dataset can be well-fitted by

$$\ln(L_i(\delta\boldsymbol{\theta})) \approx \ln(L_0) + \frac{4\|\delta\boldsymbol{\theta}\|^2}{F_i^2}, \tag{S21}$$

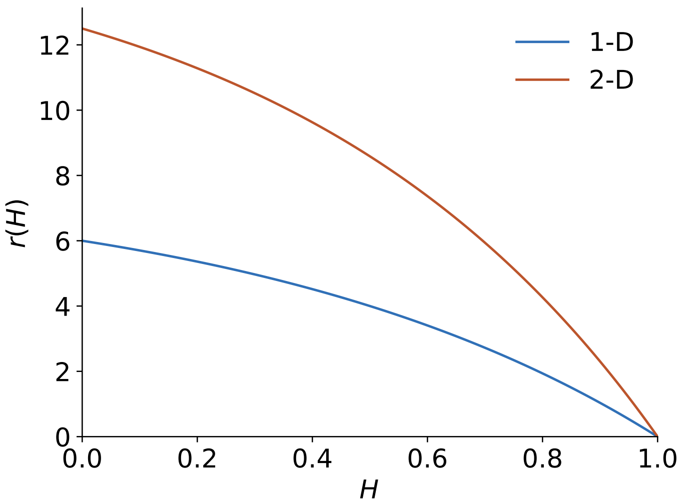

Fig. S10 Roughness coefficient decreases to zero as Hölder exponent increases to one. Lines represent the roughness coefficient $r(H) = 8 - 2^{2H+1}$ and $r(H) = 16 - 2^{2H+2} + 2^{2H} - 2^{3H-1}$ in 1-D and 2-D, respectively.

where $L_i(\delta\boldsymbol{\theta})$ is the value of the loss function at a displacement $\delta\boldsymbol{\theta}$ from the local minimum in the i-th principal component direction.

We proceed by bridging the gap between the flatness F_i, which is directional and non-local, and the total curvature index $||\mathbf{H}||_F^2$ in our analysis, which is local. We make a heuristic argument to accomplish this. Taking the second derivative of $L_i(\delta\boldsymbol{\theta})$ in equation (S21) and applying a Taylor approximation gives $(L_i)_{\delta\boldsymbol{\theta},\delta\boldsymbol{\theta}} = 8L_0/F_i^2 + \mathcal{O}(\|\delta\boldsymbol{\theta}\|^2)$. The square of this result is the i,i-derivative contribution to the total curvature at the corresponding local minimum, given by $(L_i)^2_{\delta\boldsymbol{\theta},\delta\boldsymbol{\theta}} = 64L_0^2/F_i^4 + \mathcal{O}(\|\delta\boldsymbol{\theta}\|^2)$. By analyzing the dimensions, an appropriate definition of flatness F for our analysis that retains the dimensions of F_i is:

$$F \equiv 1/\sqrt{||\mathbf{H}||_F}. \tag{S22}$$

We now consider a network that has been pre-trained for t_w iterations under a training protocol that achieves good generalization. According to the analysis of the main text, a good generalization outcome implies that a smoother basin is achieved. Assuming the smooth limit ($H \to 1^-$), we apply a Taylor approximation of the TAMSD (i.e., $\overline{\delta^2} = k\tau^{2-2H}$ for some constant k) to find the time-averaged displacement in the next (fixed) τ iterations, giving $\overline{\delta^2} \approx -2k \log \tau (H-1)$. In this limit, the expected total curvature can also be approximated as $\mathbb{E}[||\mathbf{H}||_F^2] \approx C_n(H-1)$ where C_n is a constant that depends on the dimensionality. As a result, we determine the relation:

$$\mathbb{E}[||\mathbf{H}||_F^2] \approx \frac{-C_n}{2k\log\tau}\overline{\delta^2}, \tag{S23}$$

from which we conclude that the time-averaged displacement varies with flatness as $\overline{\delta^2} \sim F^{-4}$.

8 Derivation of the FLE

In this section, we demonstrate that GD with large learning rates can be effectively approximated by the overdamped fractional Langevin equation (FLE). Our approach

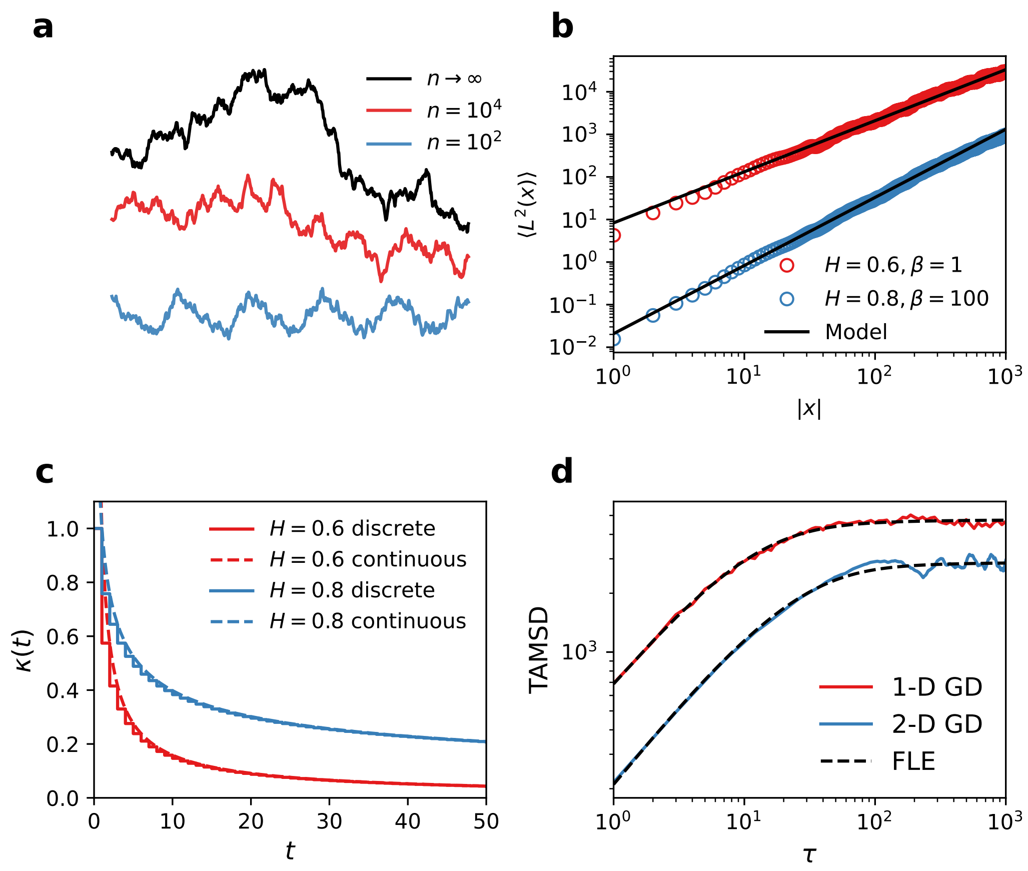

Fig. S11 Overdamped FLE accurately models GD dynamics with large learning rates on our landscape model. a, The loss function in equation (S26) approaches our landscape model as the number of scales, n, approaches infinity. **b**, The empirical variance of this loss function ($n = 10^4$) across 100 realizations agrees with our landscape model. **c**, The fractional Gaussian noise, which arises under the assumption of large learning rates, exhibits long-range correlations that induce a discrete memory kernel (equation (S50)). This discrete memory kernel (solid lines) can be approximated by the continuous power-law kernel of the Caputo fractional derivative (dashed lines), resulting in the overdamped fractional Langevin equation. **d**, The overdamped fractional Langevin equation accurately predicts the anomalous diffusive dynamics of large learning-rate GD on our landscape model ($H = 0.6$). Further examples are shown in Fig. 6 of the main text.

is inspired by recent results on the stochastic-like chaotic dynamics of deterministic GD with large learning rates [25]. Specifically, these results suggest that in a multiscale loss landscape, the gradients of small-scale components can be approximated by random noise under sufficiently large learning rates. This was proven theoretically under stringent technical conditions but validated empirically under more relaxed conditions [25]. Our analysis consists of three major steps. First, we introduce a loss function with multiple n scales that converges to our landscape model in the limit of many scales. Second, we approximate the gradients of small-scale components using the fractional Gaussian noise. We emphasize that this approximation is contingent on the assumption of large learning rates. Through a statistical mechanics approach [34], we demonstrate that fractional Gaussian noise exhibits a power-law memory effect in the dynamical equation. This effectively approximates the Caputo fractional derivative, resulting in the overdamped fractional Langevin equation (FLE). Finally, we experimentally confirm that the FLE accurately models the anomalous diffusive dynamics of GD with large learning rates in our minimal model, thus experimentally validating the assumptions of our analysis.

We begin by expressing the GD map as:

$$x_{k+1} = x_k + v_k, \tag{S24}$$
$$v_k = -\eta \nabla L(x_k). \tag{S25}$$

To analyze GD dynamics on our landscape model, we assume an n-scale loss function L that is equivalent to a fractional Brownian surface in the limit of many scales. Specifically, L takes the expression (in 1-D for tractability):

$$L(x) = \sqrt{\frac{\Delta\omega}{\beta}} \sum_{j=1}^{n} \epsilon_j f(\epsilon_j) \left[A_j \left(\cos \frac{x}{\epsilon_j} - 1 \right) - B_j \sin \frac{x}{\epsilon_j} \right], \tag{S26}$$

where $\epsilon_j \equiv 1/\omega_j$ denotes a random scale parameter, $\omega_j \sim U(n^{-a}, n^{-a}+n^b)$, $a, b \in (0,1)$, β denotes an overall scaling, $\Delta\omega = n^{a-1}$, $f^2(\epsilon) = \frac{2}{\pi}\Gamma(1-\alpha)\sin\left(\frac{\pi\alpha}{2}\right)\epsilon^{1-\alpha}$, $\alpha \in (0,1)$, and $A_j, B_j \sim \mathcal{N}(0,1)$. Since ω_j is i.i.d. uniformly distributed, ϵ_j is i.i.d. power-law distributed with probability density function:

$$p(\epsilon) = \begin{cases} n^{-b}\epsilon^{-2} & \text{for } (n^{-a}+n^b)^{-1} \le \epsilon \le n^a \\ 0 & \text{otherwise.} \end{cases} \tag{S27}$$

We numerically verify that L indeed approaches the fractional Brownian surface as $n \to \infty$ in Figure S11(a-b); for mathematical proof, see Theorem 3.2 of [35]. As highlighted in Supplementary Sec. 1, the fractional Brownian surface can be considered a local description of our multifractal landscape through the local asymptotic self-similarity property (equation (S4)). As a result, we are able to determine a relationship between the anomalous diffusive exponent α of GD dynamics and the pointwise Hölder exponent H of the multifractal landscape.

With the above choice of the loss function, equation (S25) becomes:

$$v_k = -\sqrt{\frac{\Delta\omega}{\beta_{\text{eff}}}} \sum_{j=1}^{n} f(\epsilon_j)\left(A_j \sin\frac{x_k}{\epsilon_j} + B_j \cos\frac{x_k}{\epsilon_j}\right), \tag{S28}$$

where $\beta_{\text{eff}} = \eta^{-2}\beta$. In the infinite-scales limit:

$$\lim_{n\to\infty} v_k = -\frac{1}{\sqrt{\beta_{\text{eff}} H(2H-1)}} \xi_H(x_k) \equiv -\zeta_H(x_k), \tag{S29}$$

where $H = 1 - \alpha/2$ is the pointwise Hölder exponent and ξ_H denotes the fractional Gaussian noise. The main results of [25] imply that the small-scale components of the loss gradient can be approximated by a random variable at each iteration when the learning rate is sufficiently large (i.e., $\eta \gtrsim \epsilon_j$ for all j). However, an issue with causality arises if $\xi_H(x_k)$ was directly replaced with a time-dependent fractional Gaussian noise $\xi_H(t)$. In particular, the step at any iteration would be influenced by gradients at subsequent iterations due to the long-range correlations of $\xi_H(t)$, i.e., $\lim_{n\to\infty}\langle v_{k_0} \nabla L(x_{k_0+k})\rangle = -\langle \zeta_H(k_0)\zeta_H(k_0+k)\rangle/\eta \neq 0$. Here, $\langle\cdot\rangle$ denotes the ensemble average with respect to A_j, B_j. Consistent with previous treatments of the generalized Langevin equation in statistical mechanics [34], an additional systematic

term is required to counteract this correlation. This is achieved by approximating equation (S25) with a (discrete-time) dynamical equation containing a memory term:

$$\sum_{j=0}^{k} \kappa(k+1-j)v_j - \zeta_H(k) = 0, \tag{S30}$$

where $\kappa(\cdot)$ is a memory kernel whose exact form will be determined.

We note that the condition, $\eta \gtrsim \epsilon_j$ for all j, physically expresses that the learning rate is large relative to all scales. Since the range of scales increases infinitely as $n \to \infty$, this condition would imply an unphysically large learning rate in this limit. Thus, it is relevant to consider the regime where the learning rate is large, but smaller than the largest scale(s) of the loss function. In this scenario, we assume that there exists a hidden potential function $V(x)$ that accounts for the large-scale components that do not contribute to the stochastic term (e.g., $\epsilon_j \gtrsim \eta$). As a result, equation (S30) is modified by an external force $\eta\nabla V(x)$ in the left-hand side. Consistent with this assumption, numerical simulations reveal plateaus in the TAMSD curves (e.g., Fig. S11(d)), indicating the eventual confinement of the GD optimizer within a basin. To briefly summarize the key outcome of the analysis so far, equation (S30) resembles a discrete version of the generalized Langevin equation. The memory-dependent friction force effectively arises from long-range correlations in the structure of the loss landscape. The absence of an inertial term (i.e., zero mass) implies dynamics in the overdamped regime.

We now derive the memory kernel following a statistical mechanics approach [34]. Specifically, we analytically relate the memory kernel to the autocorrelation of the fractional Gaussian noise via the fluctuation dissipation relations (FDRs). Since the interacting case (i.e., $\nabla V(x) \neq 0$) does not add anything to the derivation, we assume $\nabla V(x) = 0$ for now, reintroducing it at a later stage. By evaluating equation (S30) at time $k_0 + k$, we find that:

$$R(k_0+k;k_0) \equiv \sum_{j=0}^{k_0-1} \kappa(k_0+k+1-j)v_j - \zeta_H(k_0+k) = -\sum_{j=k_0}^{k_0+k} \kappa(k_0+k+1-j)v_j, \tag{S31}$$

where $R(k_0+k;k_0)$ is the part of equation (S30) at time k_0+k that is uncorrelated with v_{k_0} for $k>0$. It contains the random force and the component of the systematic force that depends on steps prior to v_{k_0}. As a result,

$$0 = \langle R(k_0+k;k_0)v_{k_0}\rangle \tag{S32}$$

$$= \sum_{k=1}^{\infty} \exp(i\omega k) \sum_{j=k_0}^{k_0+k} \kappa(k_0+k+1-j)\langle v_{k_0} v_j\rangle \tag{S33}$$

$$= \sum_{k=0}^{\infty}\sum_{j=0}^{k} \kappa(k+1-j)\langle v_0 v_j\rangle \exp(i\omega k) - \kappa(1)\langle v_0^2\rangle. \tag{S34}$$

Through a change of variables, $m = k+1-j$, we find that:

$$0 = \sum_{j=0}^{\infty}\sum_{m=1}^{\infty} \kappa(m)\langle v_0 v_j\rangle \exp(i\omega(j+m-1)) - \kappa(1)\langle v_0^2\rangle \tag{S35}$$

$$= \kappa(\omega) \sum_{j=0}^{\infty} \langle v_0 v_j \rangle \exp(i\omega j) - \kappa(1)\langle v_0^2 \rangle, \tag{S36}$$

where $\kappa(\omega) \equiv \sum_{k=1}^{\infty} \kappa(k) \exp(i\omega(k-1))$ is the Laplace transform of the memory kernel. Therefore,

$$\sum_{j=0}^{\infty} \langle v_0 v_j \rangle \exp(i\omega j) = \frac{\kappa(1)\langle v_0^2 \rangle}{\kappa(\omega)}, \tag{S37}$$

which is the first FDR for equation (S30).

We now analyze equation (S30) using a discrete Fourier-Laplace transform:

$$\sum_{\omega=0}^{\infty} \mathcal{F}\left\{ \sum_{j=1}^{k+1} \kappa(k+1-j) v_j \right\}(\omega) \exp(-i\omega k) = \sum_{\omega=0}^{\infty} \zeta_H(\omega) \exp(-i\omega k), \tag{S38}$$

where

$$\mathcal{F}\left\{ \sum_{j=0}^{k} \kappa(k+1-j) v_j \right\}(\omega) = \sum_{k=0}^{\infty} \sum_{j=0}^{k} \kappa(k+1-j) v_j \exp(i\omega k) \tag{S39}$$

$$= \sum_{j=0}^{\infty} \sum_{m=1}^{\infty} \kappa(m) v_j \exp(i\omega(m+j-1)) \tag{S40}$$

$$= \sum_{j=0}^{\infty} v_j \exp(i\omega j) \sum_{m=1}^{\infty} \kappa(m) \exp(i\omega(m-1)) \tag{S41}$$

$$= v(\omega)\kappa(\omega), \tag{S42}$$

and $v(\omega) \equiv \sum_{j=0}^{\infty} v_j \exp(i\omega j)$. Thus,

$$v(\omega) = \frac{\zeta_H(\omega)}{\kappa(\omega)}. \tag{S43}$$

The autocorrelation function $R_v(k) \equiv \langle v_{k_0} v_{k_0+k} \rangle$ is even by stationarity, i.e., $R_v(k) = R_v(-k)$. By the Wiener-Kinchin theorem:

$$\langle v(\omega) v(-\omega) \rangle = \sum_{k=-\infty}^{\infty} R_v(k) e^{i\omega k} \tag{S44}$$

$$= 2\Re\left\{ \sum_{k=0}^{\infty} R_v(k) \exp(i\omega k) \right\} - R_v(0) \tag{S45}$$

$$= 2\Re\left\{ \sum_{k=0}^{\infty} \langle v_0 v_k \rangle \exp(i\omega k) \right\} - \langle v_0^2 \rangle \tag{S46}$$

$$= \frac{2\kappa(1)\langle v_0^2 \rangle}{\kappa(\omega)} - \langle v_0^2 \rangle, \tag{S47}$$

Direct substitution of equation (S43) into equation (S47) shows that:

$$\langle \zeta_H(\omega) \zeta_H(-\omega) \rangle = \langle v_0^2 \rangle \left(2\kappa(1)\kappa(\omega) - \kappa(\omega)^2 \right). \tag{S48}$$

Using the equipartition theorem, $\langle v_0^2 \rangle = 1/(2\beta_{\text{eff}})$, and solving for $\kappa(\omega)$:

$$\kappa(\omega) = \kappa(1) \pm \sqrt{\kappa(1)^2 - 2\beta_{\text{eff}}\langle\zeta_H(\omega)\zeta_H(-\omega)\rangle}. \tag{S49}$$

Assuming small $\beta_{\text{eff}} \equiv \eta^{-2}\beta$, corresponding to a large learning rate and large fluctuations in the landscape, and taking the negative solution so that $\kappa(k)$ and $\langle\zeta_H(0)\zeta_H(k)\rangle$ correctly decrease as k increases, then:

$$\kappa(\omega) \approx \frac{\beta_{\text{eff}}}{\kappa(1)}\langle\zeta_H(\omega)\zeta_H(-\omega)\rangle. \tag{S50}$$

This is the second FDR for equation (S30). The unique inversion is

$$\kappa(k) = \frac{\beta_{\text{eff}}}{\kappa(1)}\langle\zeta_H(0)\zeta_H(k)\rangle. \tag{S51}$$

To determine the autocorrelation $\langle\zeta_H(0)\zeta_H(k)\rangle$, we observe that the scaled fractional Gaussian noise $\zeta_H(k)$ is exactly equal to the limit $\lim_{n\to\infty}\zeta_{H,n}(k)$, where

$$\zeta_{H,n}(k) = -\sqrt{\frac{\Delta\omega}{\beta_{\text{eff}}}}\sum_{j=1}^{n} f(\epsilon_j)(A_j \sin\frac{k}{\epsilon_j} + B_j \cos\frac{k}{\epsilon_j}). \tag{S52}$$

The proof of this limit is analogous to showing that L approaches the fractional Brownian surface in the infinite-scales limit (see Theorem 3.2 of [35]). Then,

$$\begin{aligned}
\langle\zeta_H(0)\zeta_H(k)\rangle &= \lim_{n\to\infty}\langle\zeta_{H,n}(0)\zeta_{H,n}(k)\rangle && \text{(S53)}\\
&= \lim_{n\to\infty}\frac{1}{\beta_{\text{eff}}}\sum_{j=1}^{n} f^2(\omega_j)\cos(\omega_j k)\Delta\omega && \text{(S54)}\\
&= \frac{1}{\beta_{\text{eff}}}\int_0^\infty f^2(\omega)\cos(\omega k)d\omega && \text{(S55)}\\
&= \frac{1}{\beta_{\text{eff}}|k|^\alpha}. && \text{(S56)}
\end{aligned}$$

Explicitly reintroducing the potential term, equation (S30) becomes:

$$\sum_{j=0}^{k}\frac{v_j}{|k+1-j|^\alpha} = -\eta\nabla V(x) + \frac{\eta}{\sqrt{\beta H(2H-1)}}\xi_H(t), \tag{S57}$$

which resembles a discrete version of the overdamped FLE.

Finally, to facilitate the analysis of theoretical properties [36, 37], we approximate the discrete-time dynamical equation by the continuous-time overdamped FLE. To accomplish this, we treat $v(t)$ as a step function with intervals of unit time, allowing the approximation of the sum by the Caputo fractional derivative:

$$\sum_{j=0}^{k}\frac{v_j}{|k+1-j|^\alpha} = \sum_{m=1}^{k+1}\frac{v_{k+1-m}}{m^\alpha} \tag{S58}$$

$$= \int_0^{k+1} \frac{v(k+1-m)}{m^\alpha} dm + R_k \tag{S59}$$

$$= \int_0^t \frac{v(t-t')}{t'^\alpha} dt' + R_k \tag{S60}$$

$$= \int_0^t \frac{v(t')}{(t-t')^\alpha} dt' + R_k \tag{S61}$$

$$= \Gamma(1-\alpha)\, {}^C D_t^\alpha x + R_k. \tag{S62}$$

This approximation of the memory kernel is visualized in Fig. S11(c). The local error of this approximation is:

$$R_k = \sum_{m=1}^{k+1} v_{k+1-m} \left[\frac{1}{1-\alpha}(m^{1-\alpha} - (m-1)^{1-\alpha}) - m^{-\alpha} \right], \tag{S63}$$

which converges as $k \to \infty$. Overall, we achieve the continuous-time overdamped FLE (equation (10) in the main text):

$$\Gamma(1-\alpha)\, {}^C D_t^\alpha x = -\eta \nabla V(x) + \frac{\eta}{\sqrt{\beta H(2H-1)}} \xi_H(t), \tag{S64}$$

under the assumption that GD with a large learning rate occurs within our landscape model.

We now numerically confirm that the continuous-time FLE accurately approximates the dynamics of GD on our landscape model at large learning rates, thus validating the assumptions of our analysis. To accomplish this, we demonstrate its ability to capture the crucial anomalous diffusive dynamics. Assuming the optimizer is confined in a harmonic basin, the theoretical TAMSD at large waiting times can be determined analytically:

$$\overline{\delta^2}(t_w, \tau) \approx 2\langle x_\infty^2 \rangle \left[1 - E_{2-2\mathcal{H}} \left(-\frac{\eta\lambda\tau^{2-2\mathcal{H}}}{\Gamma(2\mathcal{H}-1)} \right) \right], \tag{S65}$$

where $\langle x_\infty^2 \rangle = \eta/\beta\lambda$ is the variance of the equilibrium Gibbs distribution for a harmonic potential. We numerically verify this theoretical prediction, along with the equilibrium distribution and ensemble-average mean squared displacement (EAMSD), in Fig. S12. We also note that, for a general potential V, the plateau value is still double the variance of the equilibrium Gibbs distribution. To compare the overdamped FLE and GD, we experimentally calculate the TAMSD averaged with a window of size $T = 10^4$ over 10^3 trials of GD with $\eta = 10$ on a landscape with $H = 0.6$ and $\beta = 1$ (Fig. S11(d)). The TAMSD is calculated at a large waiting time $t_w = 10^3$ to ensure the optimizer is confined in a basin. As a result, the TAMSD exhibits a characteristic plateau. We fit the curvature parameter λ and the variance $\langle x_\infty^2 \rangle$ separately, since we find that $\langle x_\infty^2 \rangle \neq \eta/\beta\lambda$ in our experiments, suggesting the potential V is non-harmonic. Indeed, as discussed in the main text, the non-harmonic structure of V can give rise to complex non-stationary anomalous diffusion, e.g., transient super-diffusion arises in a tilted washboard-like potential. Through this general fitting procedure, we find that the empirical and theoretical TAMSD exhibit agree well for GD on our landscape model in terms of the anomalous diffusive behavior. We emphasize that this agreement is non-trivial because anomalous diffusive behavior varies significantly between different fractional dynamics [37].

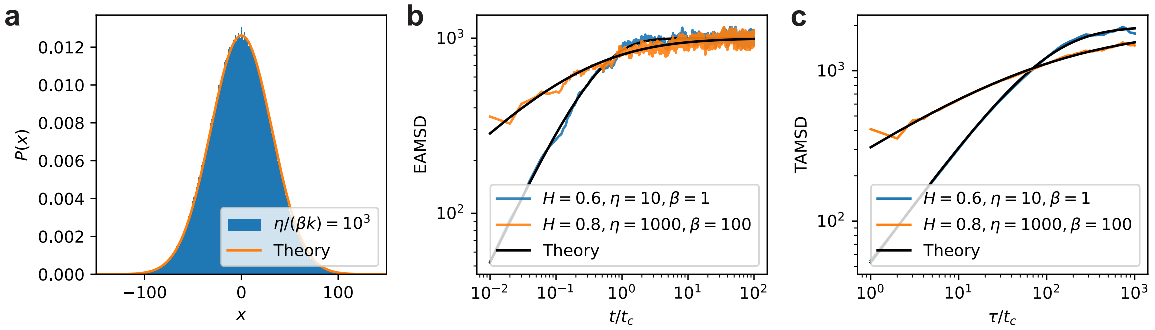

Fig. S12 Theoretical predictions of the overdamped FLE and their validation through numerical simulation. a, Using 10^2 trials of 10^4-iteration trajectories, the empirical equilibrium distribution matches the predicted Gibbs equilibrium distribution with variance $\langle x_\infty^2 \rangle \equiv \lim_{t\to\infty} \langle x^2(t) \rangle = \eta/\beta\lambda$. **b**, The ensemble-averaged mean squared displacement, given by EAMSD $= \langle x_\infty^2 \rangle \left[1 - E_\alpha(-\eta\lambda t^\alpha/\Gamma(1-\alpha))\right]$, agrees well with the numerical simulations. **c**, 10^2 simulations of 10^4-iteration trajectories shows that the empirical TAMSD ($t_w = 0, T = 10^3$) agrees well with theory, TAMSD $= 2\langle x_\infty^2 \rangle \left[1 - E_\alpha(-\eta\lambda\tau^\alpha/\Gamma(1-\alpha))\right]$. We non-dimensionalize times by dividing by a characteristic timescale $t_c = [\Gamma(1-\alpha)/(\eta\lambda)]^{1/\alpha}$.

In summary, we have theoretically demonstrated that GD with a large learning rate on our multifractal landscape model can be effectively approximated by the overdamped FLE, with experimental evidence supporting this derivation. In particular, the overdamped FLE accurately captures the anomalous diffusive dynamics of GD operating in the large learning-rate settings, which, as discussed extensively in the main text, coincides with phenomena such as an extended edge of chaos. The overdamped FLE offers a continuous-time model of the discrete-time dynamics, aligning with a common strategy to facilitate the analytic tractability of various theoretical properties of iterative optimization algorithms [38–41]. Here, we uncover fractional formalisms from the underlying correlation structure of the multifractal landscape. There is a substantial body of research supporting the appropriateness of such a fractional framework to describe anomalous diffusion driven by temporally correlated dynamics (e.g., see [29, 37] and references therein). These analytic insights could potentially be extended to understand optimization processes in other complex geometries.

9 Further evidence for fractional diffusion theory

As stated in the main text, Theorem 2 of [36] applies at the end of training where we assume that the GD optimizer is effectively confined within a limiting basin that can be modeled by a harmonic potential $\lambda\theta^2/2$. Notably, the theorem predicts the power spectral density of the limiting process (with non-dimensionalization of the FLE) as:

$$\mathcal{F}[h(\tau)] = \frac{2\eta^2\Gamma(2\mathcal{H}-1)\sin(\mathcal{H}\pi)|\omega|^{1-2\mathcal{H}}}{\beta|\Gamma(2\mathcal{H}-1)(i\omega)^{2-2\mathcal{H}} + \eta\lambda|^2}, \tag{S66}$$

where $\mathcal{F}[\cdot]$ is the Fourier transform operator, $h(\tau) \equiv \langle \theta_\infty(t)\theta_\infty(t+\tau)\rangle$ is the covariance function of the limiting process, $\mathcal{H} = \max\{1/2, H\}$, Γ is the gamma function and ω is the frequency. Note that the power spectral density represents the temporal correlations of GD learning dynamics in the Fourier domain. For large ω, the power spectral density asymptotically behaves as $\mathcal{F}[h(\tau)] \sim 1/\omega^{3-2\mathcal{H}}$. To further validate our fractional diffusion theory, we verify this asymptotic behavior in our model. We calculate $\mathcal{F}[h(\tau)]$ for the last 1000 iterations of 3000-iteration sample paths of GD

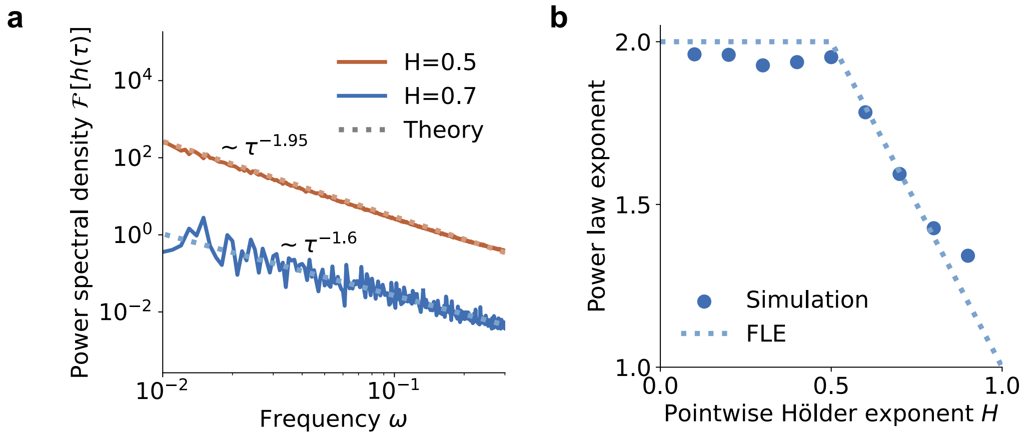

Fig. S13 Power spectral density. **a**, Examples of power spectral densities for GD dynamics in the limiting basin of loss landscapes constructed from fractional Brownian surfaces with global Hölder exponents $H \in \{0.5, 0.7\}$. Only the motion parallel to the principal component direction is used. Power laws are fitted, and their exponents are labeled. **b**, Power law exponents are similarly determined for sample trajectories on loss landscapes with global Hölder exponents $H \in \{0.1, 0.2, 0.3, \ldots, 0.9\}$. The dotted curve represents the theoretical prediction derived from the fractional diffusion theory.

on loss landscapes L constructed from fractional Brownian motions with constant $H \in \{0.1, 0.2, 0.3, \ldots, 0.9\}$. Since L is 2-D while the fractional diffusion theory was formulated in 1-D, we assume the GD trajectory can be resolved along principal components. We compute $h(\tau)$ for components of displacements in the first principal component direction and, subsequently, take the Fourier transform to obtain $\mathcal{F}[h(\tau)]$. Figure S13(a) shows examples of these power spectral densities for $H = 0.5$ and $H = 0.7$, along with their power law fits. Note that the power spectral density corresponding to the second principal component direction is not included as it is essentially a noisier version of the result for the first principal component direction; nonetheless, the fit itself is near identical. Figure S13(b) shows the result of repeating the procedure across $H \in \{0.1, 0.2, 0.3, \ldots, 0.9\}$, illustrating good agreement between our model and the fractional diffusion theory.

10 Control study: variants to loss landscape structure

As a control study, we investigate the dynamics of GD on various modifications of the loss landscape model. We note that there have been efforts to relate the structure of the loss landscape to data characteristics and network architecture. For example, visualizations have shown that deeper networks exhibit more "chaotic" loss landscapes [20], and multiscale training data can cause multiscale loss landscapes [25]. However, a precise relationship among these factors remains unclear. Thus, we consider a range of variants to the loss landscape, including monofractal, random, two-scale, and highly degenerate structures. We construct the monofractal loss landscape from a fractional Brownian surface B_H with constant $H = 0.7$. We note that other values of H yield similar results, except that the anomalous diffusion exponent at small τ changes according to $\alpha(H) = \min\{1, 2 - 2H\}$. The random loss landscape consists of uniformly distributed random values (i.e., $L(x, y) \sim U(0, 1)$ for all grid points (x, y)) on a 1024×1024 Cartesian grid. As usual, bilinear interpolation and symmetric boundary conditions are applied in these cases (see Methods). Since the random landscape is rather contrived, we also analyze models previously considered in the

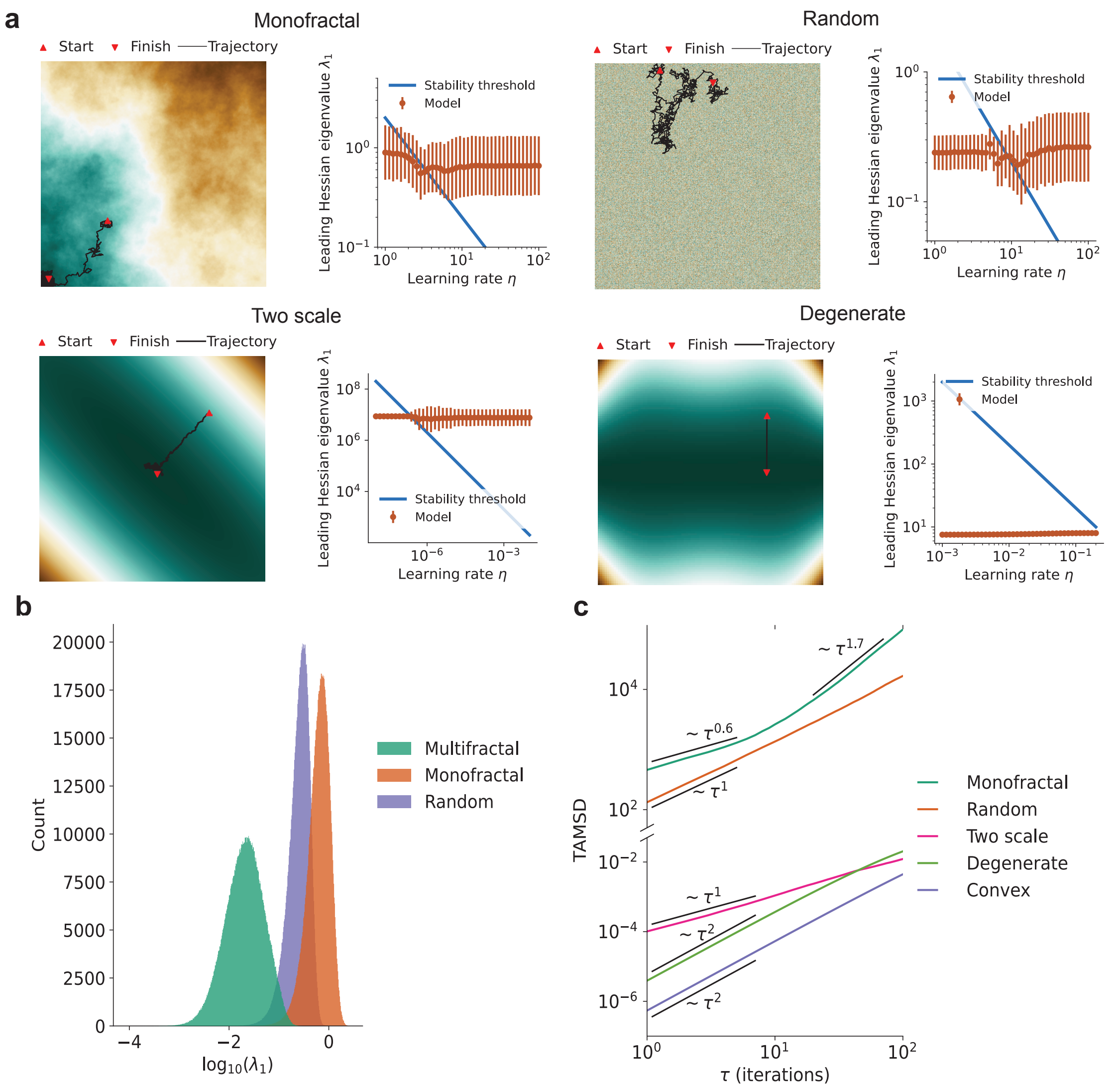

Fig. S14 GD Dynamics on variants of loss landscape structure. **a**, For monofractal ($H = 0.7$), random, two-scale and highly degenerate loss landscape structures, we visualize representative GD trajectories (left) and the leading Hessian eigenvalue λ_1 achieved for a range of learning rates η (right). The analysis is identical to the main text, except that 1000 iteration trajectories are used. **b**, The leading Hessian eigenvalue λ_1 at all Cartesian grid points for multifractal, monofractal and random loss structures. To not obscure these results, the distribution of λ_1 for the two-scale and highly degenerate landscapes are not shown. They are concentrated with significantly higher peaks around $\log_{10}(\lambda_1) \approx 1$ and $\log_{10}(\lambda_1) \approx 7$, respectively. **c**, The TAMSD ($t_w = 0$, $T = 900$) of 1000 iteration GD trajectories on each landscape variant, shown in **a**. For comparison to the highly degenerate case, the TAMSD for GD on a convex paraboloid $L(x, y) = x^2 + y^2$ is also shown. Eye-guides are provided to indicate the diffusion exponent of a power-law segment.

machine learning literature, specifically the two-scale loss landscape described in the appendix of [25] and the highly degenerate loss landscape from [42]. The two-scale loss is given by $L(x, y) = f_0(x, y) + f_{1,\epsilon}(x, y)$, where $f_0(x, y) = 0.26(x^2 + y^2) + 0.48xy$ and $f_{1,\epsilon}(x, y) = \epsilon \sin(x/\epsilon) + \epsilon \cos(y/\epsilon)$, $\epsilon = 10^{-7}$. The highly degenerate loss is given by $L(x, y) = \frac{1}{2}\epsilon\lambda_0 x^2 + \frac{1}{2}\lambda(x)y^2$, where $\lambda(x) = \lambda_0 + \lambda_1 x^2 + \lambda_2 x^4$, $\epsilon = 10^{-3}$, $\lambda_0 = 8$, $\lambda_1 = -2$, $\lambda_2 = 1$. Note that the latter was used to study SGD, where the effect of minibatch sampling was represented by a random shift of the loss landscape. We do not apply random shifts since our focus is on the dynamics of GD. We perform the same set of analyses on these variants as for our multifractal model. To summarize,

we find that only the multifractal loss landscape exhibits the EoS phenomenon and non-stationary anomalous diffusion.

To parallel our investigation of GD on a multifractal loss landscape in the main text, we simulate GD in a wide range of η for each landscape variant. Figure S14(a) shows representative sample trajectories for each loss landscape and the relationship between the learning rate η and the terminal value of the leading Hessian eigenvalue λ_1. Contrasting the multifractal loss landscape in the main text, the leading Hessian eigenvalue does not hover above the stability threshold, $2/\eta$, for a wide range of learning rates in these landscape variants (i.e., the edge of stability is not extended). Specifically, in the highly degenerate loss landscape, which is nearly convex, the optimizer diverges rapidly once η exceeds the stability threshold. Due to the ensuing numerical overflow, we measure λ_1 only for learning rates smaller than the stability threshold $\eta < 2/\lambda_1$ (i.e., within the stable convergence regime). In contrast, the two-scale structure exhibits chaotic dynamics for any practical learning rate. Only when η is extremely small does the optimizer converge monotonically to a local minimum of the small-scale structure.

We find that a key reason why the EoS phenomenon is extended in the multifractal loss landscape, but not in others, is due to its greater heterogeneity. In particular, Fig. S14(b) shows that a wider range of leading Hessian eigenvalues λ_1 is available across the local minima of a multifractal landscape compared to monofractal and random landscapes. Thus, in the multifractal landscape, there exist local minima with λ_1 just above $2/\eta$ for a wider range of η. In contrast, the reduced heterogeneity of the two-scale and degenerate landscapes, in terms of the supported values of λ_1, can be analyzed analytically. In the two-scale landscape, the Hessian has diagonal components whose values are dominated by the small-scale structure (i.e., of order ϵ^{-1}), resulting in a sharp peak of values at approximately $\lambda_1 \sim \epsilon^{-1} = 10^7$. In the highly degenerate landscape, the optimizer follows a near-zero valley along $y = 0$ toward the origin, which is the global minimum. Thus, the leading Hessian eigenvalue achieved is given by $\lambda_1 = \lambda(0) = 8$.

Furthermore, Fig. S14(c) shows the TAMSD of a representative trajectory in each loss landscape model. As predicted by the fractional diffusion theory, GD in the monofractal landscape exhibits anomalous diffusion, including sub-diffusion with a fixed exponent. In contrast, diffusive dynamics are not as diverse in the other structures. The random loss landscape exhibits only normal diffusion ($\alpha = 1$). The two-scale loss landscape can exhibit normal diffusion and super-diffusion if it is initialized sufficiently far from the origin, since it is driven by the underlying large-scale structure, but it does not yield any sub-diffusion. The degenerate structure exhibits ballistic motion ($\alpha = 2$) because its dynamics are similar to that of GD in a convex well (e.g., paraboloid) in the sense that it travels along (near) straight paths, first towards $y = 0$ then towards the origin. As visualized in Fig. S14(c), ballistic motion is characteristic of GD in a convex landscape. In summary, only the multifractal loss landscape exhibits both the EoS phenomenon and non-stationary anomalous diffusion.

11 Control study: changing the pointwise Hölder exponent

Since the spatial variation of roughness in actual loss landscapes is unknown, we now conduct a control study to determine the general applicability of our analysis on the

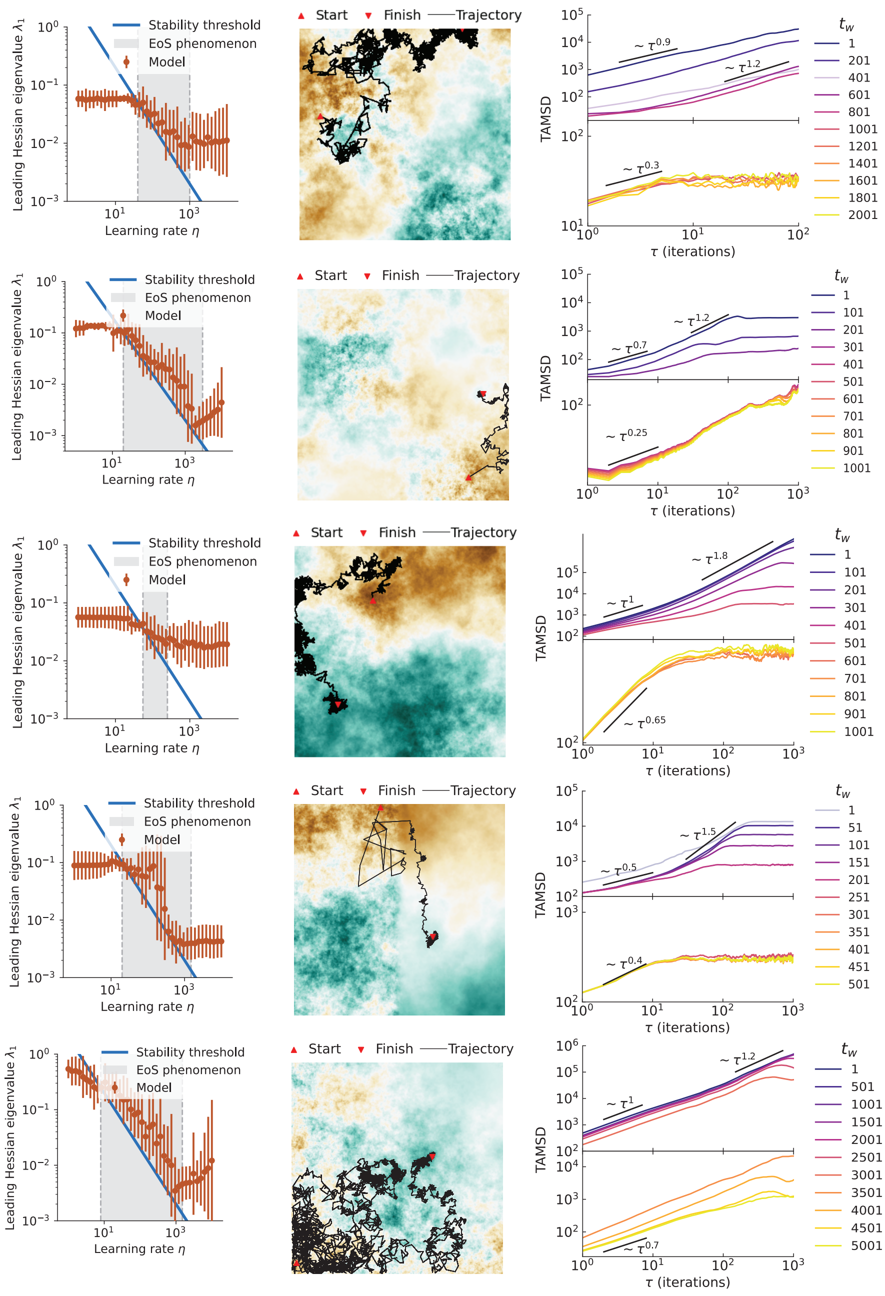

Fig. S15 Changing the pointwise Hölder exponent. On several variations to the pointwise Hölder exponent, we measure the leading Hessian eigenvalue λ_1 achieved for a range of learning rates η (left), display a sample 5000-iteration trajectory (middle), and calculate its TAMSD (right). **a**, $H(x, y) = 0.5 + 0.2\sin(\pi x/512)\cos(3\pi y/2048)$, which is the same function as the main text but the multifractal landscape realization is different. **b**, $H(x, y) = 0.5 + 0.3\sin(\pi x/512)\cos(3\pi y/2048)$, which contains a larger range of exponent values. **c**, $H(x, y) = 0.5 + 0.1\sin(\pi x/512)\cos(3\pi y/2048)$, which contains a smaller range of exponent values (smaller range of values). **d**, $H(x, y) = 0.3 + 0.4/(1 + \exp(-100(x/1024 - 0.5)))$ which is a logistic function describing variations in roughness. **e**, $H(x, y) = 0.1 + 0.8(x/1024)(y/1024)$ which is a bilinear function describing variations in roughness.

dynamics of GD in multifractal loss landscapes with different pointwise Hölder exponents. To achieve this, we generate multiple realizations of multifractal loss landscapes where the pointwise Hölder exponents vary spatially as periodic functions $H(x, y) = 0.5+0.2\sin(\pi x/512)\cos(3\pi y/2048)$ (same as main text but different realization of the multifractal landscape), $H(x, y) = 0.5+0.3\sin(\pi x/512)\cos(3\pi y/2048)$ (larger range of values, $0.2 \leq H(\theta) \leq 0.8$), and $H(x, y) = 0.5+0.1\sin(\pi x/512)\cos(3\pi y/2048)$ (smaller

range of values, $0.4 \leq H(\theta) \leq 0.6$). We also consider variations described by a logistic function $H(x, y) = 0.3 + 0.4/(1 + \exp(-100(x/1024 - 0.5)))$ and a bilinear function $H(x, y) = 0.1 + 0.8(x/1024)(y/1024)$. Figure S15 shows, for each multifractal landscape, the same analysis as in the main text. Specifically, in all examples, we find the presence of non-stationary anomalous diffusive dynamics that can be separated into a small t_w transient super-diffusive regime and large t_w sub-diffusive regime. We also find the EoS phenomenon and an extended edge of chaos. In multifractal landscapes with a broader range of pointwise Hölder exponent values, the range of learning rates η for which the leading Hessian eigenvalue λ_1 hovers above the stability threshold $2/\eta$ is larger. This result suggests that multifractality is mechanistically related to the EoS phenomenon. Overall, the dynamical properties explained in the main text are robust to changes in the pointwise Hölder exponent.

12 Relationship with existing studies of training dynamics

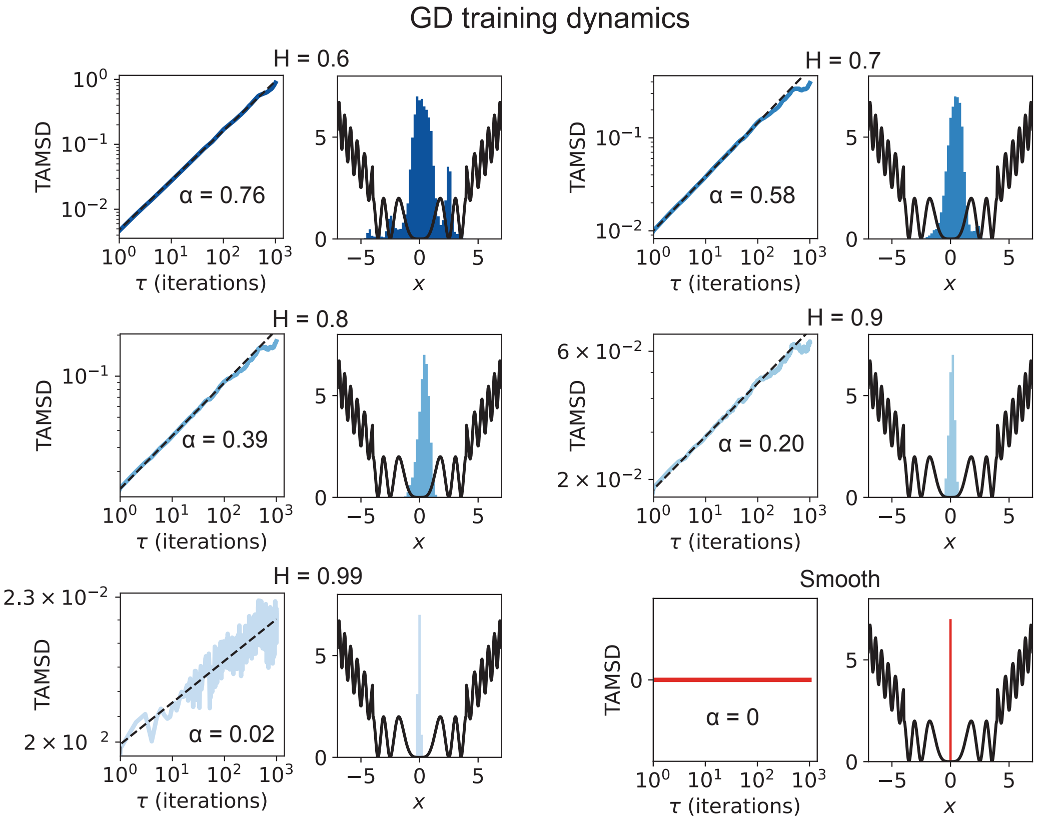

Fig. S16 GD training dynamics TAMSD ($t_w = 0$, $T = 10^4$) and histograms of 10^5 iterations of GD training dynamics (fractional Langevin equation) applied to a non-convex loss function (black solid line). The diffusion exponent α of the best power-law fit (black dashed line) is labeled.

Our study shows that a landscape-dependent annealing-like behavior can emerge from the interaction between the dynamics of deterministic GD and the roughness of the loss landscape. This mechanism allows GD to find smoother basins that house flatter minima. In contrast, previous studies on training dynamics have focused on the advantages of various forms of SGD noise for generalization, including Gaussian [38, 39], heavy-tailed [41], or multiplicative noise [43]. Landscape-dependent SGD noise has

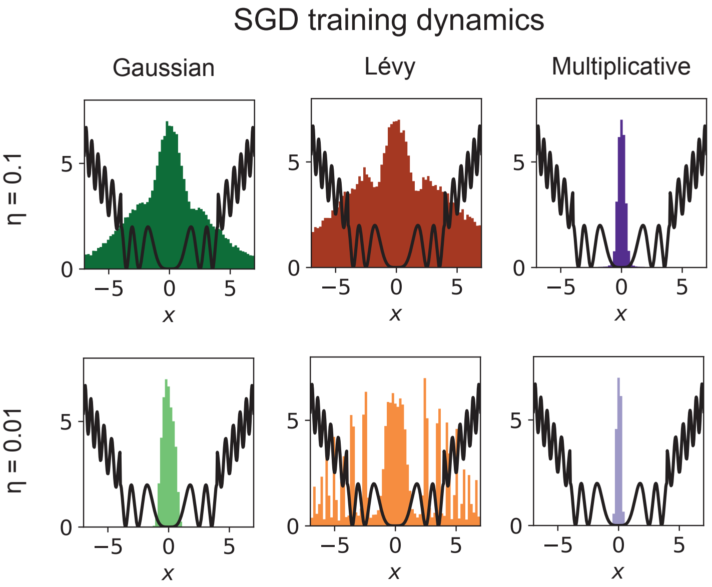

Fig. S17 SGD training dynamics Histograms of 10^5 iterations of SGD training dynamics applied to a non-convex loss function (black solid line) using various theoretical models.

been suggested to serve as an annealing strategy to find flatter minima [33]. However, there are no previous results that relate non-equilibrium training dynamics to spatially varying roughness.

To help illustrate the differences between the current study and prior studies, in this section we conduct a variation of the numerical experiment as proposed in [43]. Specifically, we simulate various training dynamics on a non-convex toy loss function with many minima, $L(x) = g(x) + 1 - \cos(x^2)$, where $g(x) = 0$ if $|x| \leq 4$ and $g(x) = x^2/10$ if $|x| > 4$ (the discontinuity at $x = \pm 4$ is not important). First, we simulate multiple sample realizations of fractional Langevin dynamics (equation 10 in the main text) with a fixed inverse temperature $\beta = 0.01$ and learning rate $\eta = 0.01$, but with varying Hölder exponent H. By simulating the fractional Langevin equation, we are effectively assuming that L is perturbed by fluctuations whose roughness is characterized by the Hölder exponent H.

The histograms in Fig. S16 show that exploration (broad searching) and exploitation (concentrated searching) can be achieved by varying the Hölder exponent H of the fractional Langevin dynamics without changing the learning rate. Specifically, as H increases towards the smooth limit ($H \to 1$), the exploitation of the central basin improves without lowering the learning rate. Since we have found that H relates to the roughness of the loss landscape at the optimizer, GD can exhibit stronger exploration ability in rough regions, which then relaxes to stronger exploitation in smooth regions. This represents a landscape-dependent annealing-like strategy implicit in GD navigating a multifractal loss landscape.

Figure S16 further demonstrates that anomalous diffusion, as opposed to normal diffusion, is central to facilitating this annealing-like behavior of GD in a multifractal loss landscape. In particular, anomalous sub-diffusion ($\alpha < 1$) occurs for GD training dynamics with $H > 0.5$; the diffusion exponent agrees well with the prediction $\alpha = 2 - 2H$ from the fractional diffusion theory. The diffusion exponent reflects the rate at which the optimizer navigates the loss landscape. Thus, as H approaches 1 and

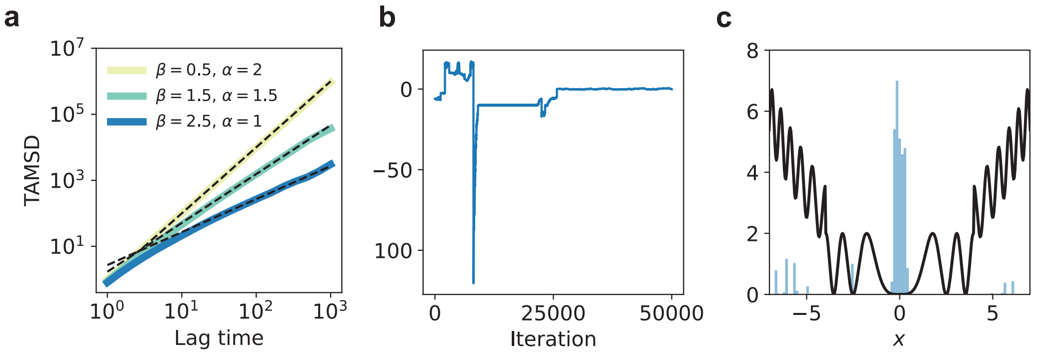

Fig. S18 Algorithm applying landscape-dependent anomalous diffusion. **a**, TAMSD of sample Lévy walk processes with various tail indexes β. Dashed lines reflect theoretical predictions of asymptotic anomalous diffusion exponents α, which are $\alpha = 2$ for $0 < \beta < 1$, $\alpha = 3 - \beta$ for $1 < \beta < 2$ and $\alpha = 1$ for $\beta > 2$. **b**, 50,000-iteration trajectory of the proof-of-concept algorithm applied to the toy loss function. The starting point is $x = -6$. **c**, Histogram of the iterates in **b**.

α approaches 0, the optimizer maintains closer proximity to the central basin, which is reflected in the histograms. Note that another way to see this effect of anomalous diffusion is through the escape time distribution. From equation 17 of the main text (ignoring the specifics of the setup of the metastability analysis), the escape time is power-law distributed with an exponent proportional to $\alpha = 2 - 2H$. This implies a higher probability of longer escape times when the diffusion exponent is smaller.

For comparison, we simulate various models of SGD, including perturbed GD with isotropic Gaussian noise (i.e., the regular Langevin equation), heavy-tailed Lévy noise and multiplicative noise. Adopting the notation of [43], these models are written as

$$\theta_{t+1} = \theta_t - \eta(L'(x) + (1+\sigma)Z_t), \tag{S67}$$

where $Z_t \sim \mathcal{N}(0,1)$ for Gaussian noise and Z_t is i.i.d. symmetric α-stable distributed for Lévy noise, and

$$\theta_{t+1} = \theta_t - \eta((1+\sigma Z_t^{(1)})L'(x) + Z_t^{(2)}), \tag{S68}$$

where $Z_t^{(1)}, Z_t^{(2)} \sim \mathcal{N}(0,1)$ for multiplicative noise. Here, σ is the noise strength, which we keep constant at $\sigma = 5$. We consider learning rates of $\eta = 0.1$ and $\eta = 0.01$. For the Lévy noise, we use a fixed stability parameter $\alpha = 1.5$.

Figure S17 reveals differences in the mechanism by which these existing models of training dynamics facilitate the finding of solutions. Notably, GD in a multifractal loss landscape finds favorable solution spaces by automatically adapting the anomalous diffusive behavior to the roughness of the loss landscape (without changing the learning rate). In contrast, existing models of SGD training dynamics with additive Gaussian or Lévy noise require the fine-tuning or annealing of the learning rate to reduce basin hopping and improve the resolution of individual basins [39, 41]. We note that learning rate fine-tuning appears less important under the assumption of multiplicative noise as it better balances exploration and exploitation than additive noise (see [43] for detailed analysis of the dynamical properties of multiplicative noise).

13 A proof-of-concept algorithm

A key insight from our results is that landscape-dependent anomalous diffusion is a powerful mechanism to target desirable solution spaces. To demonstrate that this insight can be used to inform the development of improved training algorithms, we construct a simple proof-of-concept algorithm that applies landscape-dependent anomalous diffusion as a heuristic. As detailed in Supplementary Sec. 6, anomalous diffusion can arise from several origins. Here, we propose an algorithm that induces anomalous diffusion through extreme events (that is, the Noah effect) [29]. In particular, we perturb vanilla GD with heavy-tailed noise based on an underlying Lévy walk process, which is a classic example of the Noah effect [44]. We note that the advantages of heavy-tailed noise in SGD have been analyzed, although not through the lens of anomalous diffusion [41, 43]. The Lévy process is characterized by increments of length $v\tau$ in randomly selected directions, where v is a constant speed and τ is a random waiting time whose probability density function is heavy-tailed $\psi(\tau) \sim \tau^{-1-\beta}$ with tail index $\beta > 0$. After each waiting period of ballistic motion, another direction and τ is randomly sampled. The (asymptotic) anomalous diffusion exponent of the Lévy process is $\alpha = 2$ for $0 < \beta < 1$, $\alpha = 3 - \beta$ for $1 < \beta < 2$ and $\alpha = 1$ for $\beta > 2$. Figure S18(a) shows the TAMSD of the Lévy walk process for various tail indexes β. These diverse anomalous diffusive dynamics make the process suitable for including non-stationary behavior.

We incorporate landscape-dependent anomalous diffusion by adapting the parameter β of the Lévy process based on a geometrical property of the loss landscape. Since methods of estimating roughness (e.g., pointwise Hölder exponent) are currently inefficient for deep learning contexts, as they require the sampling of several points, we adapt β to a curvature metric λ of the loss landscape (e.g., leading Hessian eigenvalue, top-k eigenvalue sum, etc.) such that $\beta = f(\lambda)$ for a suitable function $f : \mathbb{R} \to (0, \infty)$. Furthermore, since GD operates in discrete time, we round τ down to the nearest integer and use a jump-first model of the Lévy process [44] (i.e., GD with a heavy-tailed perturbation of length $v\tau$ followed by $\lfloor \tau \rfloor$ iterations of non-perturbed GD). Alg. 1 provides the pseudo-code for the algorithm.

We test the algorithm on the same toy loss function as Supplementary Sec. 12. In this example, we choose $\beta = f(\lambda)$, where $f : \mathbb{R} \to (1, 3)$ is defined by $f(\lambda) = 3 - 2(1 + \exp(10 - |\lambda|))^{-1}$ and $\lambda = \frac{d^2L}{dx^2}$. Thus, normal diffusion occurs when λ is sufficiently small, and super-diffusion with exponent $\alpha = 2(1 + \exp(10 - |\lambda|))^{-1}$ occurs when λ is sufficiently large. Figures S18(b-c) shows a sample optimization trajectory of the algorithm and the corresponding histogram of iterates. In general, we find that the optimizer initially takes rare but large steps due to fluctuations characteristic of a super-diffusive Lévy walk, which is advantageous for basin hopping. However, once the optimizer reaches the central basin (a region of low curvature), it remains there for an extended period by taking smaller, more regular steps. We find that these results are quite robust to the choice of hyperparameters, including the learning rate η and the Lévy walk speed v. Compared to the dynamical properties of SGD, which have been discussed under various assumptions about its noise structure (see Supplementary Section 12 and [43] for further details), our algorithm balances exploration and exploitation better than methods with landscape-independent additive noise. Specifically, it can escape basins more efficiently than Gaussian noise and stay within

desired basins more effectively than heavy-tailed noise. Thus, the implementation of landscape-dependent anomalous diffusion as a heuristic enables the optimizer to target desired solution spaces (flatter regions in this case) through an annealing-like strategy, demonstrating its potential applicability for the development of improved algorithms. To target solution spaces with specific geometrical properties (e.g., smoothness), we suggest that future research should focus on efficiently calculating these metrics to facilitate their practical implementation in deep learning settings.

Algorithm 1 Algorithm with adaptive anomalous diffusion

Input: Loss function $L : \mathbb{R}^n \to \mathbb{R}$, learning rate η, speed of Lévy walk process v

```
Initialize weights θ_0, t = 0;
Initialize waiting time τ = 0;
while not converged do
    if τ is zero then
        Compute curvature metric λ and shape parameter β = f(λ);
        Sample τ from Pareto distribution with shape parameter β;
        Sample random direction vector u from the unit sphere;
        Compute increment of Lévy walk process, l_t = vτu;
        Round τ to the nearest integer;
    else if τ is not zero then
        Set l_t = 0
        τ = τ − 1
    end if
    Compute gradient ∇L(θ_t)
    Update weights θ_{t+1} = θ_t − η∇L(θ_t) + l_t
    t = t + 1
end while
return θ_t
```

Supplementary References

[1] Ayache, A., Cohen, S. & Véhel, J. L. The covariance structure of multifractional brownian motion, with application to long range dependence. *2000 IEEE International Conference on Acoustics, Speech, and Signal Processing. Proceedings (Cat. No. 00CH37100)* **6**, 3810–3813 (2000).

[2] Peltier, R.-F. & Véhel, J. L. *Multifractional Brownian motion: definition and preliminary results.* Ph.D. thesis, INRIA (1995).

[3] Herbin, E. From n parameter fractional brownian motions to n parameter multifractional brownian motions. *The Rocky Mountain Journal of Mathematics* 1249–1284 (2006).

[4] Benassi, A., Roux, D. & Jaffard, S. Elliptic gaussian random processes. *Revista Matemática Iberoamericana* **13**, 19–90 (1997).

[5] Cohen, S. in *From self-similarity to local self-similarity: the estimation problem* (eds Dekking, M., Véhel, J. L., Lutton, E. & Tricot, C.) *Fractals* 3–16 (Springer, London, 1999).

[6] Lopes, R. & Betrouni, N. Fractal and multifractal analysis: A review. *Medical Image Analysis* **13**, 634–649 (2009).

[7] Ayache, A. *The generalized multifractional Brownian motion can be multifractal* (Université de Toulouse. Laboratoire de Statistique et Probabilités [LSP], 2000).

[8] Stanley, H. E. & Meakin, P. Multifractal phenomena in physics and chemistry. *Nature* **335**, 405–409 (1988).

[9] Mandelbrot, B. B. & Mandelbrot, B. B. *The fractal geometry of nature* Vol. 1 (WH freeman New York, 1982).

[10] Yang, Y. *et al.* Taxonomizing local versus global structure in neural network loss landscapes. *Advances in Neural Information Processing Systems* **34**, 18722–18733 (2021).

[11] Draxler, F., Veschgini, K., Salmhofer, M. & Hamprecht, F. Essentially no barriers in neural network energy landscape. *International Conference on Machine Learning* 1309–1318 (2018).

[12] Choromanska, A., Henaff, M., Mathieu, M., Arous, G. B. & LeCun, Y. The loss surfaces of multilayer networks. *Artificial Intelligence and Statistics* 192–204 (2015).

[13] Auffinger, A., Arous, G. B. & Černỳ, J. Random matrices and complexity of spin glasses. *Communications on Pure and Applied Mathematics* **66**, 165–201 (2013).

[14] Echelard, A., Véhel, J. L. & Barrière, O. Terrain modeling with multifractional brownian motion and self-regulating processes. *Computer Vision and Graphics: International Conference, ICCVG 2010, Warsaw, Poland, September 20-22,*

2010, Proceedings, Part I 342–351 (2010).

[15] Jastrzebski, S. *et al.* On the relation between the sharpest directions of dnn loss and the sgd step length. *arXiv preprint arXiv:1807.05031* (2018).

[16] Keskar, N. S., Mudigere, D., Nocedal, J., Smelyanskiy, M. & Tang, P. T. P. On large-batch training for deep learning: Generalization gap and sharp minima. *arXiv preprint arXiv:1609.04836* (2016).

[17] Dinh, L., Pascanu, R., Bengio, S. & Bengio, Y. Sharp minima can generalize for deep nets. *International Conference on Machine Learning* 1019–1028 (2017).

[18] Pesquet-Popescu, B. & Véhel, J. L. Stochastic fractal models for image processing. *IEEE Signal Processing Magazine* **19**, 48–62 (2002).

[19] Jaffard, S., Lashermes, B. & Abry, P. in *Wavelet leaders in multifractal analysis* (eds Qian, T., Vai, M. I. & Xu, Y.) *Wavelet analysis and applications* 201–246 (Springer, 2007).

[20] Li, H., Xu, Z., Taylor, G., Studer, C. & Goldstein, T. Visualizing the loss landscape of neural nets. *Advances in Neural Information Processing Systems* **31** (2018).

[21] Xie, T. *et al.* Evaluating loss landscapes from a topology perspective. *arXiv preprint arXiv:2411.09807* (2024).

[22] Zhang, Z., Li, Y., Luo, T. & Xu, Z.-Q. J. Stochastic modified equations and dynamics of dropout algorithm. *arXiv preprint arXiv:2305.15850* (2023).

[23] Brooks, T., Pope, D. & Marcolini, M. Airfoil Self-Noise. UCI Machine Learning Repository (1989). DOI: https://doi.org/10.24432/C5VW2C.

[24] Van der Maaten, L. & Hinton, G. Visualizing data using t-sne. *Journal of Machine Learning Research* **9** (2008).

[25] Kong, L. & Tao, M. Stochasticity of deterministic gradient descent: Large learning rate for multiscale objective function. *Advances in Neural Information Processing Systems* **33**, 2625–2638 (2020).

[26] Herrmann, L., Granz, M. & Landgraf, T. Chaotic dynamics are intrinsic to neural network training with SGD. *Advances in Neural Information Processing Systems* **35**, 5219–5229 (2022).

[27] Cvitanovic, P. *et al.* Chaos: classical and quantum. *ChaosBook. org (Niels Bohr Institute, Copenhagen 2005)* **69**, 25 (2005).

[28] Metzler, R., Jeon, J.-H., Cherstvy, A. G. & Barkai, E. Anomalous diffusion models and their properties: non-stationarity, non-ergodicity, and ageing at the centenary of single particle tracking. *Physical Chemistry Chemical Physics* **16**, 24128–24164 (2014).

[29] Vilk, O. *et al.* Unravelling the origins of anomalous diffusion: from molecules to migrating storks. *Physical Review Research* **4**, 033055 (2022).

[30] Siegle, P., Goychuk, I. & Hänggi, P. Markovian embedding of fractional superdiffusion. *EPL* **93**, 20002 (2011).

[31] Barkai, E., Garini, Y. & Metzler, R. Strange kinetics of single molecules in living cells. *Physics Today* **65**, 29–35 (2012).

[32] Bouchaud, J.-P. Weak ergodicity breaking and aging in disordered systems. *Journal de Physique I* **2**, 1705–1713 (1992).

[33] Feng, Y. & Tu, Y. The inverse variance–flatness relation in stochastic gradient descent is critical for finding flat minima. *Proceedings of the National Academy of Sciences* **118**, e2015617118 (2021).

[34] Balakrishnan, V. Fluctuation-dissipation theorems from the generalised langevin equation. *Pramana* **12**, 301–315 (1979).

[35] Kupferman, R. Fractional kinetics in kac–zwanzig heat bath models. *Journal of Statistical Physics* **114**, 291–326 (2004).

[36] Li, L., Liu, J.-G. & Lu, J. Fractional Stochastic Differential Equations Satisfying Fluctuation-Dissipation Theorem. *Journal of Statistical Physics* **169**, 316–339 (2017).

[37] Kursawe, J., Schulz, J. & Metzler, R. Transient aging in fractional brownian and langevin-equation motion. *Physical Review E* **88**, 062124 (2013).

[38] Mandt, S., Hoffman, M. & Blei, D. A variational analysis of stochastic gradient algorithms. *International Conference on Machine Learning* 354–363 (2016).

[39] Jastrzebski, S. *et al.* Three factors influencing minima in SGD. *arXiv preprint arXiv:1711.04623* (2017).

[40] Zhu, X., Wang, Z., Wang, X., Zhou, M. & Ge, R. Understanding edge-of-stability training dynamics with a minimalist example. *International Conference on Learning Representations* (2022).

[41] Şimşekli, U., Gürbüzbalaban, M., Nguyen, T. H., Richard, G. & Sagun, L. On the heavy-tailed theory of stochastic gradient descent for deep neural networks. *arXiv preprint arXiv:1912.00018* (2019).

[42] Yang, N., Tang, C. & Tu, Y. Stochastic gradient descent introduces an effective landscape-dependent regularization favoring flat solutions. *Physical Review Letters* **130**, 237101 (2023).

[43] Hodgkinson, L. & Mahoney, M. Multiplicative noise and heavy tails in stochastic optimization. *International Conference on Machine Learning* 4262–4274 (2021).

[44] Zaburdaev, V., Denisov, S. & Klafter, J. Lévy walks. *Reviews of Modern Physics* **87**, 483–530 (2015).
